# Halogenated Tryptophans Improve Integrin α_V_β_6_ Affinity of Cyclic RGD Peptides and Provide Handles for Late‐Stage Derivatization

**DOI:** 10.1002/chem.71559

**Published:** 2026-08-18

**Authors:** Beate Nachtigall, Caroline Brinkmann, Jana Langosch, Anke Nieß, Norbert Sewald

**Affiliations:** ^1^ Organic and Bioorganic Chemistry, Faculty of Chemistry Bielefeld University, Universitätsstraße 25 Bielefeld Deutschland

**Keywords:** fluorescent probes, integrins, late‐stage diversification, peptides, RGD

## Abstract

Selective targeting of integrin α_V_β_6_ is of high relevance for molecular imaging and therapeutic applications, yet ligands combining high affinity with structural and functional versatility remain limited. A structure–activity relationship (SAR) study of the nonapeptide *cyclo*‐(‐Asp‐Leu‐Ala‐Phe‐d‐Pro‐NMeLys‐Phe‐Arg‐Gly‐) was performed through site‐specific substitution of phenylalanine residues by tryptophan and halogenated tryptophan derivatives, revealing a pronounced position‐dependent effect on integrin recognition. Substitution at position *i+3* relative to the RGD sequence with halotryptophans is identified as an affinity hotspot, enabling picomolar binding to α_V_β_6_. In contrast, modification at position *i+6* preserves high α_V_β_6_ affinity while providing exceptional tolerance toward structural diversification and enabling late‐stage functionalization of unprotected peptides via palladium‐catalyzed cross‐coupling reactions. This establishes *i+6* modification as a versatile platform for chemoselective derivatization without compromising affinity. Halotryptophans serve both as pharmacophores and synthetic handles. Notably, an anthracenyl‐functionalized derivative combines sub‐nanomolar α_V_β_6_ affinity with water solubility and intrinsic fluorescence, demonstrating that even bulky *π*‐systems can be accommodated at discrete positions without loss of potency. These integrin ligands are compatible with cellular applications and enable receptor‐specific detection by flow cytometry (FACS) as well as visualization by confocal fluorescence microscopy. This work defines a robust design for α_V_β_6_‐targeting functional probes for molecular imaging and fluorescence‐guided tumor detection.

## Graphical abstract

Lighting up α_V_β_6_: Halotryptophan engineering converts an RGD scaffold into a modular α_V_β_6_ ligand platform, enabling picomolar affinity and late‐stage functionalization. The resulting probes combine high selectivity with fluorescence for FACS and tumor imaging.

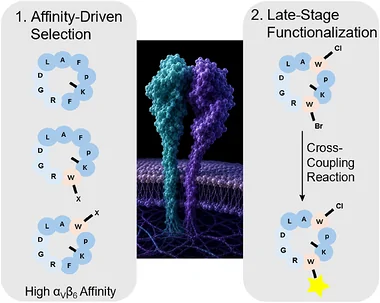

## 1 Introduction

Integrins are heterodimeric transmembrane adhesion receptors that mediate bidirectional signaling between the extracellular matrix (ECM) and the intracellular cytoskeleton. By regulating cell adhesion, migration, proliferation, apoptosis, and angiogenesis, they play central roles in tissue homeostasis and disease progression. In humans, 24 distinct noncovalent α/β heterodimers have been identified and are functionally classified into collagen‐, laminin‐, leukocyte‐, and RGD‐binding receptors [1].

The RGD‐recognizing integrins comprise eight members containing the α_V_, α_5_, α_8_, or α_IIb_ subunits. These receptors bind ECM proteins such as fibronectin and vitronectin, as well as growth factors including TGF‐β1 and TGF‐β2, via the minimal ‐Arg‐Gly‐Asp‐ (RGD) motif. Although the RGD sequence represents the common recognition element, ligand selectivity arises from differences in three‐dimensional presentation and electronic properties, which complement structurally distinct integrin binding pockets [2, 3, 4, 5, 6, 7, 8]. Since the seminal description of RGD‐mediated integrin recognition by Ruoslahti and Pierschbacher in 1984, RGD‐containing peptides and peptidomimetics have evolved into versatile targeting units for therapeutic and diagnostic applications [9, 10].

Clinically, integrin‐targeted agents have reached approval for selected indications, particularly against α_IIb_β_3_ and leukocyte integrins [11, 12]. The monoclonal antibody Abciximab inhibits platelet aggregation by blocking fibrinogen binding to α_IIb_β_3_. Among RGD‐binding integrins, α_V_‐containing receptors are strongly implicated in tumor biology. For example, α_V_β_3_ is overexpressed in neoangiogenic endothelial cells and in several malignancies. Since solid tumors typically establish their own vasculature to enable growth beyond a minimal size of approximately 2 mm in diameter, targeting and inhibiting angiogenesis emerged as a rational therapeutic strategy in cancer treatment [13]. In the oncology field, the cyclic pentapeptide Cilengitide (*cyclo*‐(‐Arg‐Gly‐Asp‐d‐Phe‐NMeVal‐), *c*‐(RGDf^(NMe)^V)) [14], developed by Kessler and coworkers, demonstrated high affinity toward α_V_β_3_ and α_V_β_5_ and advanced to Phase III clinical trials, but ultimately failed to show sufficient efficacy. Preclinical research showed that at low concentrations, Cilengitide could paradoxically stimulate angiogenesis, and dosing regimens in trials may have exposed tumors to sub‐optimal concentrations at times, complicating efficacy [15].

Cyclization of RGD peptides substantially enhances integrin affinity by restricting conformational flexibility and thereby reducing the entropic penalty upon binding. Integrin selectivity is governed by the three‐dimensional presentation of the RGD motif. While for *cyclo*‐(‐Arg‐Gly‐Asp‐d‐Phe‐Val‐) high‐affinity binding to integrin α_V_β_3_ is associated with stabilization of a βII′‐turn with d‐Phe in position *i*+1 [14, 16], its ^NMe^Val‐containing derivative Cilengitide did not display defined secondary structural features in solution, including regular β‐ or γ‐turns or stabilizing intramolecular hydrogen bonds [17].

The epithelial‐specific integrin α_V_β_6_ has emerged as a particularly attractive target in oncology and fibrosis research [18, 19]. Several viruses, for example, foot‐and‐mouth disease virus (FMDV), exploit α_V_β_6_ to attach to and enter host cells. In healthy adult tissues, its expression is minimal, whereas strong upregulation is observed in epithelial carcinomas, including lung, breast, pancreatic, prostate, ovarian, gastric, and oral cancers [20]. Elevated levels of α_V_β_6_, for example in liver and colon cancer, correlate with a poor prognosis, which underscores the importance of this receptor as a diagnostic and therapeutic target [18]. A distinguishing feature of α_V_β_6_ is its high affinity for the latency‐associated peptide of TGF‐β1 (LAP‐TGF‐β1). Binding of LAP‐TGF‐β1 to α_V_β_6_ mediates cytokine activation and thereby influences proliferation, differentiation, and immune modulation, processes that are central to tumor progression and fibrotic disease [21, 22, 23].

Structurally, the α_V_ subunit comprises a β‐propeller, thigh, and calf domains, while the β_6_ subunit contains a head region and two leg domains. Ligand binding occurs at the α_V_/β_6_ interface and involves RGD‐mediated contacts as well as coordination of the ligand aspartate to a Mg^2^^+^ ion in the metal‐ion‐dependent adhesion site (MIDAS). Like other integrins, α_V_β_6_ adopts the conserved integrin architecture composed of an α_V_ β‐propeller domain and a β_6_ βA domain forming the headpiece, followed by leg domains that allow transitions between bent (inactive) and extended (active) conformations. High‐resolution structural insight has been obtained for α_V_β_6_ in complex with the latency‐associated peptide of TGF‐β1 (LAP‐TGF‐β1), revealing how the integrin recognizes the RGD motif and mediates force‐dependent activation of latent TGF‐β1 [23].

Based on structural elements of the natural ligand, Kessler and co‐workers had designed cyclic nonapeptides that mimic the α_V_β_6_‐binding epitope [7]. These ligands, for example, *cyclo*‐(‐Asp‐Leu‐Ala‐Phe‐d‐Pro‐NMeLys‐Phe‐Arg‐Gly‐) [*c*‐(DLAFp^(NMe)^KFRG)] exhibit high nanomolar to sub‐nanomolar affinities and have served as lead structures for the development of selective α_V_β_6_‐targeting probes and conjugates. α_V_β_6_ combines disease‐selective expression, well‐characterized structural determinants of ligand recognition, and validated pharmacological tractability. These features render cyclic RGD scaffolds particularly attractive starting points for affinity optimization and late‐stage functionalization strategies, including the incorporation of halogenated amino acids to modulate binding interactions and enable downstream derivatization. This can lead to increased passive membrane permeability and enhanced resistance to proteolytic degradation [24].

Fluorescently labeled RGD peptides are valuable molecular probes for the investigation of RGD‐binding integrins, including α_V_β_3_ [25], α_V_β_6_, and α_V_β_8_. By combining receptor‐specific targeting with an optical readout, they enable quantitative analysis of integrin expression, binding affinity, and receptor‐mediated internalization in cell‐based systems. In addition to mechanistic studies, fluorescent RGD ligands serve as tools for validating target engagement of drug conjugates and nanoparticles, and they hold translational potential in molecular imaging and fluorescence‐guided surgery. Their small size, defined stoichiometry, and rapid tissue penetration provide advantages over antibody‐based probes.

Halogen substituents are well‐established modulators of molecular recognition, capable of influencing lipophilicity, steric demand, electronic distribution, and halogen bonding interactions. In peptide‐based ligands, the incorporation of halogenated amino acids can, therefore, alter affinity and selectivity toward protein targets while simultaneously providing synthetic handles for late‐stage functionalization [26, 27]. Previous studies demonstrated that halogenated tryptophans enhance binding of RGD peptides to integrin α_V_β_3_ [28]. Given the increasing biomedical relevance of integrin α_V_β_6_ in carcinoma, fibrosis, and viral pathogenesis, we sought to investigate whether analogous halogenation strategies improve affinity and selectivity of cyclic RGD peptides toward α_V_β_6_ and related integrins.

Specifically, phenylalanine residues within the established high‐affinity α_V_β_6_ ligand *c*‐(DLAFp^(NMe)^KFRG) (**0**) were replaced by regioselectively halogenated tryptophans (5‐, 6‐, and 7‐bromo‐ and chlorotryptophan). Beyond affinity optimization, brominated analogues were designed to enable late‐stage derivatization via cross‐coupling chemistry for the generation of fluorescent labels or small‐molecule drug conjugates (SMDCs).

## 2 Results and Discussion

### 2.1 Halogenated Tryptophans Improve Integrin Affinity of Cyclic RGD Peptides

Halogenated tryptophans were prepared enzymatically using flavin‐dependent halogenases (RebH, Thal, PyrH) [29]. The enzymes were applied as cross‐linked enzyme aggregates (CLEAs), enabling straightforward product isolation, recyclability, and direct Fmoc protection for solid‐phase peptide synthesis. Compared to classical electrophilic halogenation, this approach provided superior regioselectivity under mild conditions [30]. If required, d‐Tryptophan may be directly halogenated by the C5‐halogenase Thal, while other halogenated d‐Trp derivatives are obtained by dynamic stereoinversion [31]. Modified amino acids were incorporated into the cyclic nonapeptide scaffold derived from the previously established α_V_β_6_ ligand using Fmoc‐based solid‐phase peptide synthesis (SPPS) on chlorotrityl resin, followed by head‐to‐tail cyclization. Binding affinities toward α_V_β_6_, α_V_β_8_, α_V_β_3_, α_5_β_1_, and α_IIb_β_3_ were determined by competitive ELISA [4] (Tables 1 and S2–S4) and complemented by cell‐based assays (cell adhesion, Table 2, and FACS analysis, see also Table S5).

**TABLE 1:** Integrin affinity (IC_50_ values [nM]) of the modified RGD nonapeptides according to competitive ELISA (see also Tables S2–S4).

No	Peptide	α_V_β_6_	α_V_β_8_	α_V_β_3_	α_5_β_1_	α_IIb_β_3_	Selectivity α_V_β_6_/α_V_β_8_	Selectivity α_V_β_6_/α_V_β_3_
**0**	*c*‐(DLAFp^(NMe)^KFRG) (ref [7].)	0.26	23.6	632	72.9	—	91	2431
**1**	*c*‐(DLAFp^(NMe)^K^(5‐Br)^WRG)	0.23 ± 0.02	16.6 ± 3.2	> 10^5^	—	>5·10^4^	72	> 10^5^
**2**	*c*‐(DLAFp^(NMe)^K^(6‐Br)^WRG)	0.52 ± 0.09	7.5 ± 0.8	> 10^5^	—	>5·10^4^	14.4	> 10^5^
**3**	*c*‐(DLAFp^(NMe)^K^(7‐Br)^WRG)	0.68 ± 0.21	10.0 ± 0.3	> 10^5^	—	>5·10^4^	14.7	> 10^5^
**4**	*c*‐(DLAWp^(NMe)^K^(6‐Br)^WRG)	0.59 ± 0.07	66.2 ± 14.1	—	—	—	112	—
**5**	*c*‐(DLA^(5‐Cl)^Wp^(NMe)^KFRG)	4.21 ± 1.88	—	>2·10^4^	—	—	—	—
**6**	*c*‐(DLA^(6‐Cl)^Wp^(NMe)^KFRG)	3.46 ± 0.40	—	22.3 ± 1.1	—	—	—	—
**7**	*c*‐(DLA^(5‐Br)^Wp^(NMe)^KFRG)	0.083 ± 0.028	14.0 ± 0.8	242 ± 67	0.82 ± 0.85	>10^4^	169	2916
**8**	*c*‐(DLA^(6‐Br)^Wp^(NMe)^KFRG)	0.036 ± 0.009	39.7 ± 3.4	247±132	4.57 ± 0.03	>10^4^	1102	6861
**9**	*c*‐(DLA^(7‐Br)^Wp^(NMe)^KFRG)	0.24 ± 0.06	26.2 ± 9.3	38±17	0.036 ± 0.001	>10^4^	109	158
**10**	*c*‐(DLA^(6‐Br)^Wp^(NMe)^KWRG)	0.17 ± 0.01	15.3 ± 1.3	—	—	—	90	—
**11**	*c*‐(DLA^(6‐Br)^Wp^(NMe)^K^(6‐Br)^WRG)	0.51 ± 0.03	30.7 ± 2.4	—	—	—	60	—
**12**	*c*‐(DLA^(6‐Cl)^Wp^(NMe)^K^(6‐Br)^WRG)	0.35 ± 0.12	—	—	—	—	—	—

IC_50_ values were obtained by sigmoidal dose‐response fitting and were calculated as the mean values of at least three replicates. The selectivity α_V_β_6_/α_V_β_8_ is given as the ratio IC_50_ (α_V_β_8_)/IC_50_ (α_V_β_6_) and the selectivity α_V_β_6_/α_V_β_3_ is given as the ratio IC_50_ (α_V_β_3_)/IC_50_ (α_V_β_6_).

**TABLE 2:** Results of the cell adhesion assays with HN cells (α_V_β_6_+) compared to the affinities determined by a competitive ELISA (see also Tables S2–S4).

No.	Peptide	IC_50_ cell adhesion (µM)	IC_50_ ELISA (nM)
**0**	*c*‐(DLAFp^(NMe)^KFRG) (ref [7].)	22.34 ± 4.30	0.26
**7**	*c*‐(DLA^(5‐Br)^Wp^(NMe)^KFRG)	6.80 ± 2.69	0.083 ± 0.028
**8**	*c*‐(DLA^(6‐Br)^Wp^(NMe)^KFRG)	4.24 ± 1.24	0.036 ± 0.009
**9**	*c*‐(DLA^(7‐Br)^Wp^(NMe)^KFRG)	3.75 ± 0.77	0.24 ± 0.06

IC_50_ values were obtained by sigmoidal dose‐response fitting and were calculated as the mean values of at least triplicates.

The nonapeptides (Table 1) were synthesized using Fmoc‐based SPPS on chlorotrityl resin, starting with glycine as the first residue. This resin enables cleavage under mild acidic conditions, which is essential for preserving acid‐labile side‐chain protecting groups required for subsequent head‐to‐tail cyclization. Amino acid couplings were performed using DIC (diisopropylcarbodiimide) in combination with Oxyma as the coupling system and a large excess of the respective amino acids. In contrast, the halotryptophans were employed only in slight excess, using HOBt as an additive and extended reaction times to ensure efficient incorporation. Complete conversion, particularly for sterically demanding residues such as arginine and proline or for amino acids used in limited quantities, was verified by test cleavages followed by LC‐MS analysis. A capping step with acetic anhydride was performed after each coupling cycle to suppress potentially undetected free amino groups. The addition of 0.1 M HOBt in the Fmoc deprotection solution effectively minimized aspartimide formation, which was otherwise observed occasionally. Following cleavage from the resin under mild acidic conditions, the linear peptides were subjected to head‐to‐tail cyclization under pseudo‐dilution conditions [32], which was completed by cleaving the side chain protecting groups.

### 2.2 Structure–Activity Relationships Determined by ELISA

The ELISA applied here is a competitive binding assay to assess peptide–integrin interactions at the isolated receptor level. An integrin‐specific extracellular matrix ligand is immobilized on a microtiter plate. Purified integrin is then added together with the test peptide in varying concentrations, allowing competition for receptor binding. If the integrin binds to the coated ligand, it remains on the plate and is detected via an enzyme‐linked antibody, generating a color signal after substrate conversion. If the peptide inhibits binding, the integrin is washed away, resulting in reduced or absent signal [4].

Structurally, the combination of d‐proline (p) in the *i+4* position and *N*‐methyllysine (^(NMe)^K) in the *i+5* position likely enforces a stabilized β‐turn conformation, preorganizing the RGD pharmacophore for optimal receptor interaction. The additional halogen substituent may further contribute through enhanced hydrophobic contacts within the integrin binding pocket.

All modifications were classified according to their position relative to the RGD motif to systematically interpret the structure–activity relationships (SAR) of the investigated cyclic peptides. Within this framework, substitutions occur either at the *i+3* position, corresponding to the aromatic residue within the LAW segment adjacent to the RGD pharmacophore, or at the *i+6* position relative to the RGD motif. This positional distinction provides a unifying basis for understanding both affinity and selectivity trends across the series.

Substitution of phenylalanine (F) at the *i+3* position relative to aspartic acid (D) in the RGD motif proved to be the key determinant for affinity and selectivity modulation (Table 1). Introduction of 5‐ or 6‐bromotryptophan at this position (**7**, **8**) resulted in a pronounced enhancement of α_V_β_6_ affinity compared to the reference peptide *c*‐(DLAFp^(NMe)^KFRG) **0** (IC_50_ = 0.26 nM). In particular, incorporation of 6‐bromotryptophan (^(6‐Br)^W, peptide **8**) yielded the most potent analogue, *c*‐(DLA^(6‐Br)^Wp^(NMe)^KFRG), with an IC_50_ value of 0.036 nM, corresponding to an approximately sevenfold improvement over the reference compound and representing sub‐nanomolar potency. The 5‐bromo analogue (^(5‐Br)^W, peptide **7**) reached 0.083 nM, whereas the 7‐bromo derivative (^(7‐Br)^W, peptide **9**) showed slightly reduced but still high affinity (0.24 nM), indicating a clear positional effect of the halogen substituent within the indole ring.

Modifications at the *i+3* position exert the most pronounced influence on α_V_β_6_ binding. This residue seems to be highly sensitive to steric and electronic perturbations, consistent with a location deeply embedded within the integrin binding pocket [6, 23]. Introduction of halogenated Trp, particularly bromotryptophan, resulted in a substantial increase in α_V_β_6_ affinity and selectivity. Among these, substitution at the 6‐position of the indole ring proved optimal, yielding the most potent compounds of the series. This strong positional preference indicates a highly defined spatial arrangement within the binding site, where substituents must align precisely to enable favorable hydrophobic interactions. In contrast, substitution at alternative positions of Trp, such as the 5‐ or 7‐position, led to reduced affinity, underscoring the stringent geometric requirements at *i+3*, in addition to enhancing α_V_β_6_ affinity. A clear halogen effect was observed when comparing chloro‐ and bromo‐substituted analogues at the *i+3* position. In contrast to the ^(5‐Br)^W or ^(6‐Br)^W substitution (**7** and **8**), the chlorinated derivatives (^(5‐Cl)^W, peptide **5** and ^(6‐Cl)^W, peptide **6**) showed a pronounced loss of α_V_β_6_ affinity (IC_50_ = 3.46–4.21 nM). Thus, replacement of chlorine by bromine improved α_V_β_6_ affinity by approximately one to two orders of magnitude. This trend suggests that the larger atomic radius, higher polarizability, and increased hydrophobicity of bromine are crucial for productive interactions within this specific integrin binding pocket. These modifications also caused a pronounced reduction in binding to off‐target integrins, suggesting that selectivity arises not only from improved target interactions but also from steric and electronic exclusion of competing binding pockets. In contrast, modifications at the *i+6* position have a less direct impact on binding affinity and are generally better tolerated.

In addition to the high α_V_β_6_ affinity, these analogues maintained nanomolar activity toward α_V_β_8_ (14.0–39.7 nM for **7**–**9**). This translates into α_V_β_6_/α_V_β_8_ selectivity in the range of approximately 110 (peptide **9**) up to >1000 (peptide **8**), demonstrating that subtle positional halogenation can dramatically influence integrin subtype selectivity. Notably, *c*‐(DLA^(6‐Br)^Wp^(NMe)^KFRG) **8** combines the highest potency with the most pronounced selectivity over α_V_β_3_ and negligible affinity for α_IIb_β_3_ (>10^4^ nM), resulting in a selectivity window of more than four orders of magnitude versus the platelet integrin. Affinity toward α_5_β_1_ remained weak, albeit in the nM range, except for peptide **9**, which showed unexpectedly increased α_5_β_1_ binding (0.036 nM), indicating that substitution at the 7‐position may partially shift integrin preference. Although both integrins bind RGD‐containing ligands, α_V_β_3_ and α_5_β_1_ differ substantially in their physiological and pathological roles. Integrin α_V_β_3_ is minimally expressed in most quiescent adult tissues but becomes strongly upregulated in activated endothelial cells, osteoclasts, and many aggressive tumors. It is particularly associated with pathological angiogenesis, tumor invasion, and metastatic progression, making it a hallmark of disease‐associated neovascularization. In contrast, α_5_β_1_ is a principal fibronectin receptor with broad expression in normal tissues and a central role in physiological processes such as embryonic development, wound healing, matrix assembly, and mechanotransduction. Pathologically, α_5_β_1_ contributes to tumor progression by mediating tumor–stroma interactions, extracellular matrix remodeling, and cell adhesion‐mediated therapy resistance. Thus, whereas α_V_β_3_ is often considered a marker and driver of pathological angiogenesis, α_5_β_1_ represents a more ubiquitous regulator of tissue architecture whose dysregulation in cancer promotes invasion and microenvironmental adaptation rather than serving as a strictly disease‐specific target [33].

In contrast, modification at the *i+6* position (**1**–**3**), achieved by replacing the second phenylalanine of the lead structure **0** with bromotryptophan derivatives ^(5‐Br)^W, ^(6‐Br)^W, or ^(7‐Br)^W, did not improve α_V_β_6_ affinity (0.23–0.68 nM vs. 0.26 nM for peptide **0**), indicating that this region is less conformationally constrained and likely more solvent‐exposed. Interestingly, the simultaneous replacement of Phe at *i+3* with Trp and Phe at *i+6* with 6‐BrTrp (^(6‐Br)^W) maintained affinity for integrin α_V_β_6_ while substantially reducing affinity for integrin α_V_β_8_, resulting in an almost tenfold increase in the α_V_β_6_/α_V_β_8_ selectivity (cf. **2** and **4**, Table 1). While substitutions at *i+6* do not significantly enhance affinity, they can modulate overall ligand properties without disrupting the core interaction with α_V_β_6_, supporting a secondary, rather than primary, role in integrin recognition. However, these analogues exhibited dramatically reduced binding to α_V_β_3_ (IC_50_>10^5^ nM, corresponding to an improvement in selectivity of roughly two orders of magnitude. Although potency remained largely unchanged, this represents a significant design success, as the bulky halotryptophan introduces an additional functionalization site without compromising α_V_β_6_ activity. The preservation of affinity despite increased steric demand and *π*‐system expansion suggests compensatory interactions mediated by the halogen substituent. Further combination strategies (**10**–**12**) did not surpass the potency of the single ^(6‐Br)^W substitution at *i+3* (**8**), indicating that excessive steric expansion may counteract favorable conformational effects.

Importantly, across all evaluated peptides, affinity toward α_IIb_β_3_ was negligible (IC_50_ > 10^4^ nM where determined). This lack of interaction with the platelet integrin is highly relevant for prospective therapeutic applications, as it minimizes the risk of interference with hemostasis and other α_IIb_β_3_‐mediated physiological processes.

Overall, the data clearly identify halogenated tryptophan at the *i+3* position—particularly ^(6‐Br)^W—as the decisive structural modification for achieving sub‐nanomolar α_V_β_6_ potency combined with excellent subtype selectivity.

### 2.3 Cell‐Based Validation in a Competitive Adhesion Assay

The competitive ELISA experiments with isolated α_V_β_6_ integrin provided evidence for the high binding affinities of the synthesized peptides at the receptor level, revealing nanomolar IC_50_ values and distinct SAR. However, while ELISA assays allow quantification of direct ligand–receptor interactions under controlled conditions, they do not account for the complexity of the cellular context. As integrins are membrane‐embedded receptors whose binding properties can be influenced by their physiological environment, results obtained with purified proteins should be interpreted with appropriate caution. To further assess the binding affinity under more physiological conditions, a competitive adhesion assay with crystal violet staining was implemented. Validation with Cilengitide in WM115 cells gave an IC_50_ of 0.61 ± 0.08 µM, consistent with literature values, confirming assay reliability [34].

Competitive adhesion assays with fibronectin or LAP‐TGF‐β1 as natural ligands demonstrated comparable performance; due to practical considerations, fibronectin was selected for subsequent experiments. Application of the optimized assay to brominated nonapeptides (**7**–**9**) revealed micromolar IC_50_ values in cell‐based adhesion assays, despite nanomolar affinities in ELISA with isolated α_V_β_6_ (Table 2).

All modified peptides showed improved cell adhesion compared to the lead structure **0**, with up to a 10‐fold enhancement. However, discrepancies between receptor‐based and cell‐based affinities for the 7‐brominated analogue suggest that additional factors such as hydrophilicity, steric effects, or cellular processing may influence functional activity at the cellular level.

### 2.4 Fluorescently Labeled RGD Peptides

For cellular investigations, fluorescent derivatives of the lead ligands were prepared to enable direct analysis of receptor binding and internalization. Fluorophores were either introduced as fluorescent amino acids directly during solid‐phase peptide synthesis or by late‐stage functionalization of the fully assembled peptide. Halogenated residues served as orthogonal handles for late‐stage functionalization via cross‐coupling reactions (e.g., Suzuki–Miyaura or Mizoroki–Heck), enabling the in situ generation of *π*‐extended fluorescent systems directly on the peptide scaffold.

Azulenylalanine (^(Az)^A) was synthesized using tryptophan synthase [35] and incorporated at the phenylalanine position in the *i + 6* position relative to the RGD sequence, as this position was identified to be compatible with structural changes without compromising integrin binding. The ^(Az)^A‐containing peptides *c*‐(DLA^(6‐Cl)^Wp^(NMe)^K^(Az)^ARG) (**13**) and *c*‐(DLA^(6‐Br)^Wp^(NMe)^K^(Az)^ARG) (**14**) showed high affinity toward integrin α_V_β_6_ (Figure 1 and Table 3). However, compared to the phenylalanine analogues **6** and **8**, the affinity of the 5‐chlorotryptophan‐containing peptide **13** is higher, and the affinity of the 5‐bromotryptophan derivative **14** is lower. Their absorption maxima at 272 and 287 nm still fall in the UV range, which is problematic for cellular applications due to phototoxicity, strong background absorption by biomolecules, limited compatibility with standard instrumentation, and poor tissue penetration, resulting in low signal quality.

**FIGURE 1:**
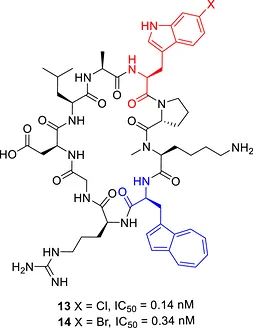
Azulenylalanine‐containing peptides **13** and **14**.

**TABLE 3:** Affinity (IC_50_ values [nM]) of fluorogenic RGD peptides to integrin α_V_β_6_ according to competitive ELISA (see also Tables S2–S4).

No	Peptide	α_V_β_6_	α_V_β_8_
**13**	*c*‐(DLA^(6‐Cl)^Wp^(NMe)^K^(Az)^ARG)	0.14 ± 0.064	—
**14**	*c*‐(DLA^(6‐Br)^Wp^(NMe)^K^(Az)^ARG)	0.34 ± 0.129	—
**15**	*c*‐(DLA^(6‐(4‐vinyl‐benzoate))^Wp^(NMe)^KFRG)	4.75 ± 0.95	—
**16**	*c*‐(DLA^(6‐(9‐anthracenyl))^Wp^(NMe)^KFRG)	10.3 ± 0.40	—
**17**	*c*‐(DLA^(5‐(1‐pyrenyl))^Wp^(NMe)^KFRG)	3.76 ± 0.38	138 ± 33
**18**	*c*‐(DLA^(5‐(3‐aminophenyl))^Wp^(NMe)^KFRG)	0.095 ± 0.016	23.7 ± 2.7
**20**	*c*‐(DLAFp^(NMe)^K^6‐(9‐anthracenyl)^WRG)	0.53 ± 0.001	—
**21**	*c*‐(DLA^(5‐Cl)^Wp^(NMe)^K^6‐(9‐anthracenyl)^WRG)	0.52 ± 0.015	—
**22**	*c*‐(DLA^(6‐Cl)^Wp^(NMe)^K^6‐(9‐anthracenyl)^WRG)	0.53 ± 0.089	—

IC_50_ values were obtained by sigmoidal dose‐response fitting and were calculated as the mean values of at least triplicates.

### 2.5 Fluorogenic Late‐Stage Modification of Bromotryptophan‐Containing Peptides by Suzuki–Miyaura or Mizoroki–Heck Cross‐Coupling

Haloarene moieties in peptides provide versatile handles for palladium‐catalyzed C─C bond‐forming reactions, including Mizoroki–Heck, Sonogashira–Hagihara, and Suzuki–Miyaura cross‐couplings [26]. These transformations enable selective late‐stage modification of peptides in protected or unprotected form. Importantly, previous studies have shown that halogenated integrin ligands can undergo such palladium‐catalyzed functionalizations without substantial loss of binding affinity [28]. This strategy therefore offers a powerful approach to diversify peptide ligands and to introduce functional groups that may facilitate the development of peptide–drug conjugates, an area of growing relevance in modern drug design.

However, the introduction of additional substituents may influence both the electronic properties and steric environment of the peptide scaffold. Accordingly, it was necessary to evaluate how such modifications affect ligand performance. Within this study, the focus was placed on Suzuki–Miyaura [28] and Mizoroki–Heck [36] cross‐coupling reactions (Figure 2). Both reactions are compatible with peptide substrates under comparatively mild conditions and allow selective functionalization of halogenated tryptophan residues while preserving the polar character and aqueous solubility of the peptides, which is essential for subsequent in vitro applications.

**FIGURE 2:**
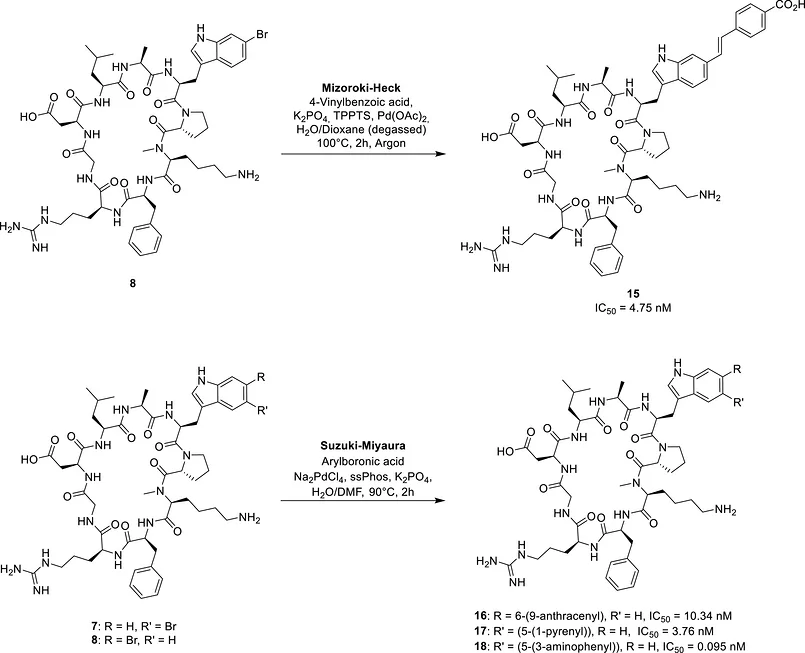
Cross‐coupling reactions on the high‐affinity peptide ligand **8** containing 6‐bromotryptophan in the *i + 3* position relative to the RGD sequence (see also Supporting Information).

The procedure, including the choice of palladium catalysts and coreagents, followed the protocol reported by Kemker and Gruß [28, 36]. All target compounds were obtained in isolable form. Notably, the solvent volume exerted a pronounced influence on the reaction time; thus, a peptide concentration of approximately 30 mmol/mL was maintained. As anticipated, compounds **15, 16,** and **17** bearing modifications at position *i+3* displayed reduced affinities relative to the parent compound **0**, but remained in the nanomolar range. While the Mizoroki‐Heck cross‐coupling of **8** with 4‐vinylbenzoic acid to give **15** was accompanied by a 130‐fold affinity loss towards α_V_β_6_ (IC_50_ = 4.75 nM), the Suzuki–Miyaura cross‐couplings of **7** or **8** with 9‐anthracenylboronic acid and 1‐pyrenylboronic acid, respectively, yielded **16** with a nearly 290‐fold lower affinity (IC_50_ = 10.34 nM) and **17** with only a 104‐fold lower affinity (IC_50_ = 3.76 nM). While **18**, bearing a 3‐aminophenyl substituent, exhibits excellent affinity to α_V_β_6_ (0.095 nM), the bulkier anthracenyl and pyrenyl groups in **16** and **17**, respectively, lead to a substantial decrease in activity. This indicates that the *i+3* position is closely involved in receptor interaction and subject to tight steric constraints. For late‐stage diversification, halogen reactivity was strategically exploited. 5‐ or 6‐chlorotryptophan residues were preferentially placed at the affinity‐relevant *i+3* position to maintain structural integrity and receptor binding, whereas the more reactive 6‐bromotryptophan was introduced at the *i+6* position as a synthetic handle (**19** and **12**). This arrangement enabled chemoselective Suzuki–Miyaura cross‐coupling on fully unprotected cyclic peptides. Due to the faster oxidative addition of C─Br compared to C–Cl bonds, selective mono‐functionalization at the brominated position was achieved while leaving the chlorinated residue practically untouched. Thus, 5‐ or 6‐chlorotryptophan at *i+3* and 6‐bromotryptophan at *i+6* establish a modular platform for affinity optimization and orthogonal late‐stage diversification of high‐affinity α_V_β_6_‐targeting ligands. The reactivities of the individual halotryptophan residues differed markedly, allowing the boronic acid to be employed in 2.4–3.0 equivalents. For the doubly 6‐halogenated peptide *c*‐(DLA^(6‐Cl)^Wp^(NMe)^K^(6‐Br)^WRG) (**12**), only minor amounts of the di‐conjugated product were detected at the end of the reaction; this could likely be further minimized by shorter reaction times (Figure 3). The side product was readily removed by chromatography, enabling the selective functionalization of a dihalogenated, unprotected peptide via cross‐coupling. The cross‐coupling reaction was also performed on the fully side‐chain‐protected peptide, that is, directly after the cyclization step, to assess a potentially more streamlined synthetic route. However, this approach proved impractical due to purification difficulties arising from limited solubility. Moreover, the side‐chain protecting groups were found to be unnecessary for the reaction and, given the high solubility of the unprotected peptides, enabled straightforward aqueous workup by preparative HPLC. For affinity comparisons, the cyclic RGD peptide containing Phe at position *i+3* (analogous to the lead structure) and 6‐bromotryptophan at position *i+6* (**2**) was also used in a Suzuki–Miyaura cross‐coupling reaction.

**FIGURE 3:**
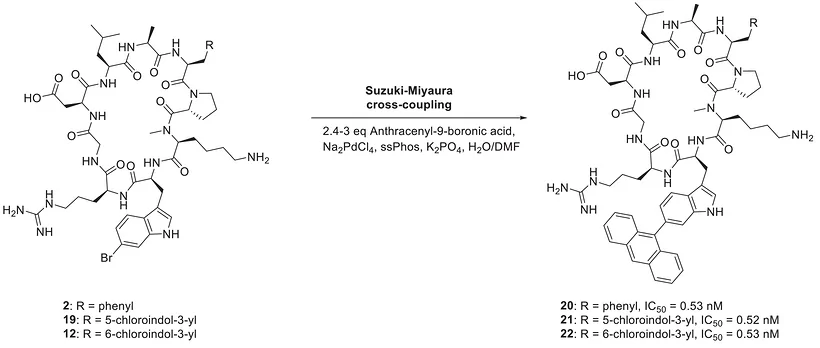
Suzuki–Miyaura cross‐coupling products were synthesized using unprotected dihalogenated peptides with highly selective coupling at the 6‐bromotryptophan position. 9‐Anthracenylboronic acid was used in excess (2.4–3 eq.). The reactions were carried out under inert conditions, and the solvents were degassed beforehand (see also Supporting Information).

The successful synthesis of compounds **20**–**22** highlights the potential of this strategy for late‐stage modification of peptidic constructs. Following chromatographic purification, the peptides were evaluated for their binding affinities (Table 3). All compounds exhibited excellent aqueous solubility.

The affinity of the three peptides with an anthracenyl label in the *i+6* position (**20**–**22**) toward α_V_β_6_ is in the same sub‐nanomolar range. This is surprising, given that the *i+3* position was found to have a greater impact on the affinity than the *i+6* position. Nevertheless, their high affinity compared to compound **16** (10.34 nM) underlines the crucial role of positional dependency when incorporating bulky fluorophores.

Importantly, the substituents introduced in this study (azulenyl, pyrenyl, anthracenyl, aminophenyl, and vinylbenzoate) are fluorophoric moieties, enabling direct optical readout. Thus, despite variations in affinity, several derivatives combine high binding potency with intrinsic fluorescence, rendering them attractive probes for fluorescence‐based cellular studies.

Overall, these results demonstrate that the *i+3* position is highly sensitive to steric and directional effects and that single modifications at this position can be detrimental if improperly oriented, while simultaneous functionalization of tryptophan at *i+3* (halogenation) and *i+6* (fluorophore attachment) enables cooperative interactions, providing access to fluorescent peptide ligands with high affinity for α_V_β_6_ integrin.

Peptides **13** and **14**, bearing azulene chromophores, show absorption maxima below 300 nm in the UV region and are therefore unsuitable for most imaging applications. Consequently, despite high α_V_β_6_ affinity, these peptides are limited as direct fluorescent probes. In contrast, anthracenyl‐containing peptides **20**, **21**, and **22** exhibit a bathochromic shift to 368 nm, representing a clear improvement due to the extended *π*‐system. Importantly, these bulky substituents are well tolerated without loss of affinity, consistent with the higher structural tolerance at position *i + 6*. However, excitation at ∼368 nm still remains suboptimal for biological imaging, and anthracene is prone to environmental quenching and photobleaching.

Notably, the use of fluorogenic amino acids represents an elegant design strategy, as it enables the direct integration of chromophores into the peptide backbone while preserving overall aqueous solubility despite the presence of highly hydrophobic aromatic systems. This intrinsic balance between hydrophobic functionality and peptide‐mediated solubility is a key advantage of the approach.

### 2.6 FACS Analysis of Peptide‐Integrin Interaction

HN cells, which exhibit high α_V_β_6_ and moderate α_V_β_8_ expression, were used as a model system to evaluate the selective binding of fluorescent α_V_β_6_‐targeting ligands by flow cytometry (FACS). Incubation at 4°C and 37°C enabled differentiation between non‐specific uptake and energy‐dependent uptake.

The anthracenyl‐modified nonapeptide **21** was selected for these studies, as it combines high α_V_β_6_ affinity with suitable optical properties (see Supporting Information), allowing excitation, for example, at 405 nm. A clear signal shift between 4°C and 37°C indicates energy‐dependent interaction and internalization, while minimal differences between untreated and cells incubated at 4°C suggest negligible nonspecific uptake (Figure 4). Despite the increased hydrophobicity introduced by the *π*‐system, no significant unspecific uptake was observed.

**FIGURE 4:**
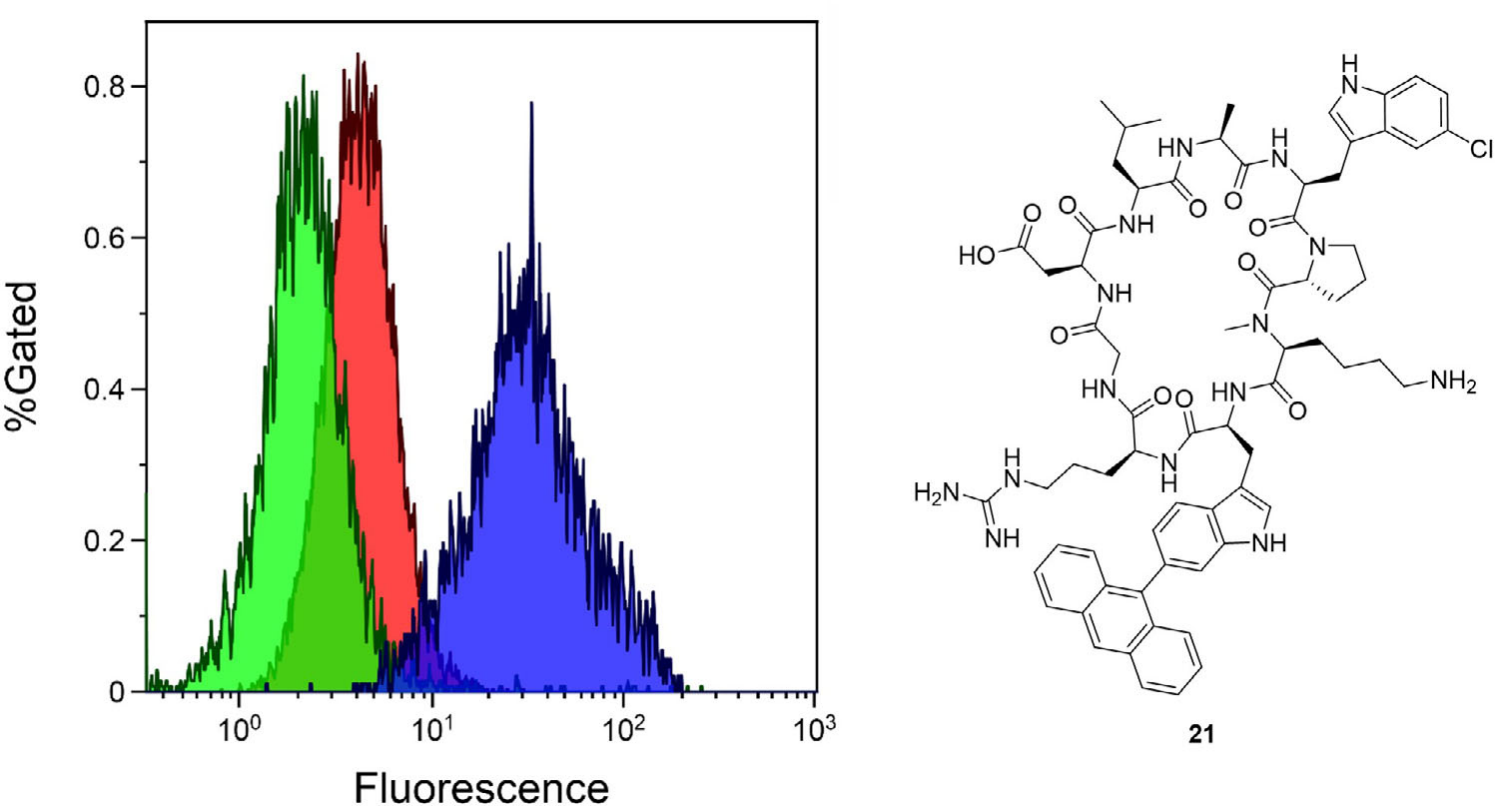
FACS measurement: green: untreated HN‐cells; red: HN‐cells treated with **21** at 4°C, 1 h; blue: HN‐cells treated with **21** at 37°C, 1 h.

## 3 Conclusion

Systematic substitution of one of the two phenylalanine residues in the parent RGD‐containing nonapeptide by tryptophan and halogenated tryptophan derivatives revealed a clear and position‐dependent SAR with respect to integrin α_V_β_6_ binding and selectivity over integrin α_V_β_3_. Within this framework, the two key positions relative to the RGD motif, *i+3* and *i+6*, play fundamentally distinct roles in governing affinity, selectivity, and functionalization potential.

Modification at position *i+6* (compounds **1**–**3**) preserves the high α_V_β_6_ affinity of the parent scaffold (IC_50_ = 0.23–0.68 nM vs. 0.26 nM for **0**), while dramatically suppressing α_V_β_3_ binding (> 10^5^ nM), resulting in exceptional selectivity (α_V_β_6_/α_V_β_3_ up to ∼5 × 10^4^). These data demonstrate that substitution at *i+6* is highly effective in enhancing subtype selectivity without compromising intrinsic binding affinity, identifying this position as a key determinant for selectivity tuning. There is no major effect of the replacement of Phe by Trp, except the affinity ratio α_V_β_6_/α_V_β_8_ (cf**. 2** and **4**). Notably, in contrast to peptides lacking bulky fluorophores, ^Az^Ala‐containing peptides in the *i+6* position exhibit higher α_V_β_6_ affinity when combined with chlorinated than brominated derivatives at *i+3*.

In contrast, modification at position *i+3* (compounds **5**–**10**) exerts a stronger influence on intrinsic α_V_β_6_ affinity. While chlorinated derivatives (**5** and **6**) show reduced potency, brominated analogues (**7**–**9**) display a marked affinity enhancement, reaching the low picomolar range (e.g., **8**: IC_50_ = 36 pM). However, this gain in affinity is accompanied by a partial loss of selectivity, as residual binding to α_V_β_3_ and other integrins becomes detectable. Nevertheless, the affinity ratio α_V_β_6_/α_V_β_3_ remains favorable. Thus, position *i +3* primarily governs affinity optimization, albeit sometimes with a trade‐off in selectivity. Simultaneous modification of both positions (**11**) does not lead to additive effects but instead reflects a delicate balance between affinity and selectivity, highlighting a non‐linear interplay between the two sites.

Beyond binding optimization, halogenated tryptophans enable late‐stage diversification of the globally deprotected peptides via palladium‐catalyzed cross‐coupling reactions such as Suzuki–Miyaura and Mizoroki–Heck reactions. Building on this SAR framework, fluorogenic amino acids were introduced to enable functional probe development, and their effects were likewise interpreted in the context of their position relative to the RGD motif. At the *i+3* position, the tolerance toward fluorogenic modification is strongly limited by steric constraints. Small aromatic extensions, such as the 3‐aminophenyl substituent, are well accommodated and maintain excellent α_V_β_6_ affinity, demonstrating that modest expansion of the indole system is compatible with binding. However, incorporation of larger aromatic fluorophores, including pyrene, anthracene, or vinyl benzoate at the *i+3* position, results in a marked loss of potency. These findings are fully consistent with the SAR of halogenated analogues and reinforce the conclusion that the *i+3* position is tightly constrained within the binding pocket, permitting only substituents that closely match the steric and electronic requirements of the site. In contrast, fluorogenic modifications introduced at the *i+6* position are significantly better tolerated. Even bulky polyaromatic systems such as anthracene retain strong α_V_β_6_ affinity, suggesting that this position is oriented toward a more solvent‐accessible region where steric constraints are relaxed.

The differential reactivity of chloro‐ and bromo‐substituted indole systems allows for chemoselective functionalization, where peptides bearing chlorotryptophan at *i+3* and bromotryptophan at *i+6* provide an ideal platform for selective derivatization at the *i+6* position. This strategy is exemplified by the introduction of extended *π*‐systems, such as 9‐anthracenyl substituents (**21** and **22**), which retain high α_V_β_6_ affinity. Notably, the latter compounds demonstrate that even bulky fluorophores can be accommodated without loss of potency, underscoring the remarkable structural tolerance at position *i+6*.

Taken together, these results reveal a clear spatial separation of function within the peptide scaffold: the *i + 3* position serves as a structurally constrained hotspot that governs binding affinity and selectivity, whereas the *i + 6* position represents a permissive site suitable for functionalization. This distinction provides a robust design principle for fluorogenic α_V_β_6_ ligands, in which minimal and precisely tuned modifications are required at *i + 3* to preserve high‐affinity binding, while bulky reporter groups can be introduced at *i + 6* to enable signal generation without compromising target recognition.

Overall, these findings establish a unified design principle for α_V_β_6_‐targeting peptides: position *i + 6* serves as the key determinant for selectivity enhancement and late‐stage functionalization, whereas position *i + 3* primarily modulates binding affinity. The resulting ligands combine sub‐nanomolar to picomolar α_V_β_6_ affinity with excellent selectivity profiles and high synthetic versatility. Given the prominent role of α_V_β_6_ as a tumor‐associated integrin, these compounds represent powerful tools for integrin detection in FACS‐based assays and confocal microscopy and hold strong potential for fluorescence‐guided tumor imaging applications.

## Conflicts of Interest

The authors declare no conflicts of interest.

## Supporting information



The authors have cited additional references within the Supporting Information [37, 38, 39, 40].
**Supporting File 1**: chem71559‐sup‐0001‐SuppMat.docx

## Data Availability

The data that supports the findings of this study are available in the Supporting Information of this article.
