# Supplementary material for: Halogenated Tryptophans Improve Integrin αVβ6 Affinity of Cyclic RGD Peptides and Provide Handles for Late‐Stage Derivatization
Source: Chemistry. 2026 Aug 18;32(41):e71559. doi: 10.1002/chem.71559 (PMC13729994; doi:10.1002/chem.71559)
Supplement: Supplementary file 1 [file CHEM-32-e71559-s001.docx]

Content

[General	2](#_Toc230770647)

[Enzymatic Halogenation via CombiCLEAs	4](#_Toc230770648)

[Halogenated Amino Acids	6](#_Toc230770649)

[Enzymatic Synthesis of Tryptophan Derivatives with Tryptophan Synthase	9](#_Toc230770650)

[Fmoc protection of tryptophan derivatives	12](#_Toc230770651)

[Peptide synthesis	16](#_Toc230770652)

[Halogenated Nonapeptides	18](#_Toc230770653)

[Late stage modification of Peptides	41](#_Toc230770654)

[Suzuki-Miyaura-coupling	41](#_Toc230770655)

[Heck-reaction	41](#_Toc230770656)

[Cell Culture and Bioassays	51](#_Toc230770657)

[Solid-phase binding assay (ELISA)	51](#_Toc230770658)

[Cell culture und used cell lines	54](#_Toc230770659)

[Flow cytometry for determination of integrin-expression levels	55](#_Toc230770660)

[Flow cytometry for investigating cellular uptake	56](#_Toc230770661)

[Crystal violet-based adhesion assay	57](#_Toc230770662)

[References	58](#_Toc230770663)

[Appendix	59](#_Toc230770664)

[NMR- and UV-Spectra	61](#_Toc230770665)

# General

All solvents were acquired from Merck, BLDPharm and Carl-Roth in the highest available grades (typically analytical grade. For all experiments highly pure water was used from an ELGA PURELAB Flex 2 system with UV and TOC monitoring. Amino acids were purchased from BLD Pharmatech GmbH. Haloindoles and boronic acids were purchased from BLD Pharmatech GmbH or Acros Organics. Dry DCM was freshly distilled from CaH_2_.

Analytical RP-HPLC was performed on a Shimadzu Nexera XR UHPLC equipped with a pump LC-20AD, an autosampler SIL-20AXR, a column oven CTO-2CA, a diode array detector SPD-M20A, and a communication module CBM-20A using a Phenomenex Luna C_18_ column (3.0 μm, 100 × 2.0 mm^2^).

Analytical LC-MS and determination of ESI-HRMS (Phenomenex Luna C_18_ column (3.0 μm, 100 × 2.0 mm^2^) were performed on an Agilent 6220 TOF-MS (Agilent Technologies) with a dual ESI source operating with a spray voltage of 2.5 kV, a 1200 HPLC system with an autosampler, a degasser, a binary pump, a column oven, and a diode array detector. Nitrogen was generated by a nitrogen generator NGM 11 and served as the nebulizer and dry gas. External calibration was performed with ESI-L tuning mix (Agilent Technologies).

Preparative HPLC was performed using a Merck-Hitachi LaChrom HPLC consisting of an interface D-7000, a pump L-7150, a detector L-7420, and a Hypersil Gold C_18_ column (1.9 μm, 250 × 21.2 mm^2^) or a Hypersil Gold C_18_ column (7 μm, 250 × 10.0 mm^2^). Alternatively, an automatic flash-chromatography Reveleris X2 (Büchi AG) equipped with a C_18_ column (Biotage, SNAP Ultra C_18_, 25 μm, 12 g) was used for purification.

NMR spectra were recorded on a Bruker DRX-500, Avance III 500 (^1^H: 500 MHz; ^13^C: 126 MHz), or Avance 600 (^1^H: 600 MHz; ^13^C: 151 MHz) spectrometer. Chemical shifts are given in parts per million (ppm) relative to tetramethylsilane; residual solvent peaks for ^1^H and ^13^C were used as the internal standard.

Accurate nano-ESI measurements are performed using a Q-IMS-TOF spectrometer Synapt G2Si (Waters) in resolution mode, interfaced to a nano-ESI ion source. Nitrogen serves both as the nebulizer gas and the dry gas for nano-ESI and is generated by the nitrogen generator NGM 11. The mass axis was internally calibrated with the protonated LeuEnk ion as internal calibration standard. Scan accumulation and data processing was performed with MassLynx 4.1 (Waters) on a PC workstation. Determination of exact masses were performed using centroid data.

Acquisition of fluorescence excitation and emission spectra was performed on a Jasco FP-8300 fluorescence spectrometer equipped with a Peltier thermostat 4-position automatic cell changer (10 mm rectangular cells) at 20 °C using a Xe light source.

UV–visible spectroscopy was performed on an Agilent 8453 system equipped with a combination of a deuterium-discharge lamp and a tungsten lamp to cover the UV–vis wavelength range from 190 to 1100 nm. As a detector, a photodiode array is used and a tempered 8-position sample holder with temperature control using a PT-104A resistance thermometer. Measurements were performed at 20 °C.

# Enzymatic Halogenation via CombiCLEAs

#### CombiCLEAs

The genes encoding *pyrH*, and *thal* were obtained from Life Technologies as codon optimized synthetic genes and subcloned as published previously.^[1]^ *RebH* was previously isolated as described by Frese et al., and has since been used in our laboratory.^[2]^

In a 300 mL Erlenmeyer flask, 50 mL of LB-medium with ampicillin (100 µg/mL) and chloramphenicol (25 µg/mL) was inoculated from glycerol cryocultures using a sterile pipette tip. The culture was grown overnight at 37 °C in an incubator shaker at 200 rpm. On the following day, 500 mL of TB-medium with ampicillin (100 µg/mL) and chloramphenicol (25 µg/mL) in 2 L baffled culture flask was inoculated with 10 mL of preculture and incubated at 37 °C to an OD_600_ of 0.6–0.8. Then the pGro7 plasmid was induced by the addition of 2 g/L of l-arabinose. Growth continued at 37 °C for 30 min before reducing the temperature to 25 °C, followed by the addition of isopropyl *β-d*-thiogalactopyranoside (IPTG) to a final concentration of 0.1 mm. After 20 h of expression at 25 °C, the culture was harvested by centrifugation (4000 × *g*, 20 min, 4 °C). The pelletized cells were resuspended in 100 mm sodium phosphate buffer (pH = 7.4) and centrifuged (14,000 × *g*, 5 min, 4 °C). The supernatant was discarded; the cells were flash-frozen in liquid nitrogen and stored at -20 °C until use.

A frozen pellet of *E. coli* BL21 (DE3) pGro7 pETDUET-FHal-ptdh-borf (FHal = thalH, pyrH, rebH) from 0.5 L of TB-culture was thawed at room temperature and then resuspended in 100 mm sodium phosphate buffer (pH = 7.4) to a 25 % suspension. 1 mg of DNAse was added. High pressure homogenization using a French pressure cell press (2 passages, 1200 bar) or cell disruption by ultrasonication (30 minutes, pulsation 5) was followed by centrifugation (12,000 × *g* for 20 min, 4 °C) to remove cell debris. The supernatant was transferred to a 50 mL centrifuge tube, and ammonium sulfate was added according to the amount given in 1. To precipitate the proteins the centrifuge tube was gently inverted a few times. The resulting suspension was gently shaken at 4°C in the incubator for one hour and then stored at 4 °C. Glutardialdehyde (50% aqueous solution) was added for crosslinking of the precipitated proteins (volumes according to 1) and the centrifuge tube was shaken for another two hours.

Table S1: Quantities for the preparation of the Halogenases combi-CLEAs. The reference quantity here is the clarified lysate.

| Halogenase | Ammonium sulfate | | 50% Glutardialdehyde | |
| --- | --- | --- | --- | --- |
|  | **% Saturation** | **mg/mL Lysate** | **mM** | **μL/ mL Lysate** |
| PyrH | 75 | 675 | 67 | 12.31 |
| RebH | 100 | 731 | 48 | 8.69 |
| Thal | 50 | 375 | 77 | 13.94 |

The immobilized proteins were separated by centrifugation (14,000 × *g*, 10 min, 4 °C) and the supernatant was discarded. The combiCLEAs were resuspended and washed three times in a 100 mM Na_2_HPO_4_ buffer. After resuspension they were added to the reaction mixture or stored at 4°C.

The resuspended combiCLEAs were added to the buffer (15 mM Na_2_HPO_4_, 30 mM NaBr or NaCl, 1 μM FAD, 100 μM NAD^+^, pH=7.4, 1.2 L) containing tryptophan (PyrH: 0.7 mM, RebH: 1.4 mM, Thal: 2mM) and equilibrated to 25 °C in an incubator shaker (Infors Minitron, 150 rpm). Conversion was monitored by analytical RP-HPLC. After complete conversion, the CLEAs were removed by centrifugation, the aqueous medium was concentrated under reduced pressure and desalted using a C18 silica gel filter column with water as the mobile phase. The halogenated tryptophan was then eluted with methanol.

# Halogenated Amino Acids

#### 5-Bromo-L-tryptophan

**^1^H-NMR** (DMSO-d6, 500 MHz) 𝛿 [ppm] = 11.24 (s, 1H, N^1^**H**), 8.12 (s, 3H, N**H**_3_^+^), 7.76 (s, 1H, C^4^**H**), 7.34 (d, ^3^*J* = 8.6 Hz, 1H, C^6^**H**), 7.27 (d, ^4^*J* = 2.4 Hz, 1H, C^2^**H**), 7.20 (d, ^3^*J* = 8.6 Hz, C^7^**H**), 4.13 (dd, ^3^*J* = 6.8 Hz, ^3^*J* = 5.8 Hz, C^α^**H**), 3.23 (dd, ^2^*J* = 15.1 Hz, ^3^*J* = 5.8 Hz, 1H, C^β^**H**), 3.17 (dd, ^2^*J* = 15.1 Hz, ^3^*J* = 6.8 Hz, 1H, C^β^**H**).

**^13^C-NMR** (DMSO-d6, 126 MHz) 𝛿 [ppm] = 170.7 (*quart.* C, 1C, **C**OOH), 134.9 (*quart.* C, 1C,**C**^7a^), 129.0 (*quart.* C, 1C, **C**^3a^), 126.5 (*tert.* C, 1C, **C**^4^), 123.6 (*tert.* C, 1C, **C**^6^), 120.7 (*tert.* C,1C, **C**^2^), 113.5 (*tert.* C, 1C, **C**^7^), 111.3 (*quart*. C, 1C, **C**^5^), 107.1 (*quart*. C, 1C, **C**^3^), 52.9 (*tert*. C, 1C, **C**^α^), 26.0 (*sek*. C, 1C, **C**^β^H2).

#### 5-Chloro-L-tryptophan

| **HR-MS** (*m/z*): | Calculated  [M+H]^+^ 239.05818 | Determined  [M+H]^+^ 239.0591 |
| --- | --- | --- |

#### 6-Bromo-L-tryptophan

**^1^H-NMR** (600 MHz, dmso-d_6_) [δ/ppm]: 11.18 (s, 1H, N**H**^indole^), 7.54 (s, 1H, C**H**^indole-7^), 7.53 (d, *^3^J* = 8.7 Hz, 1H, C**H**^indole-4^), 7.24 (s, 1H, C**H**^indole-2^), 7.09 (dd, *^3^J*= 8.4 Hz, *^4^J*= 1.7 Hz, 1H, C**H**^indole-5^), 3.46 (d, *^3^J*= 8.1 Hz, 1H, C**H**^α^), 3.26 (dd, ^2^*J*= 15.2 Hz, *^3^J*= 4.3 Hz, 1H, C**H**_2_^ß^), 3.00 (dd, ^2^*J*= 15.1 Hz, *^3^J*= 8.3 Hz, 1H, C**H**_2_^ß^).

**^13^C-NMR** (151 MHz, DMSO-d_6_) [δ/ppm]: 170.1 (1C, **C**O_2_H), 137.2 (1C, **C^q,^** ^indole^NH), 126.5 (1C, C**^γ^C^q,^** ^indole^), 125.2 (1C, **C**^indole-5^), 121.1 (1C, **C**^indole-2^), 120.3 (1C, **C**^indole^**^-^**^4^), 113.8 (1C, **C**Br), 113.6 (1C, **C**^indole^**^-^**^7^), 110.0 (1C, **C^γ^**C^ß^H_2_), 54.7 (1C, **C**^α^), 26.9 (1C, **C**^ß^H_2_)

| **ESI-MS** (*m/z*): | Calculated  [M^79Br^+H]^+^ 283.01  [M^81Br^+H]^+^ 285.01 | Determined  [M^79Br^+H]^+^ 283.02  [M^81Br^+H]^+^ 285.02 |
| --- | --- | --- |

#### 6-Chloro-L-tryptophan

**^1^H-NMR** (600 MHz, DMSO-d_6_) [δ/ppm]: 11.21 (d, *^3^J*= 2.4 Hz, 1H, N**H**^indole^), 7.58 (d, *^3^J*= 8.4 Hz, 1H, C**H**^indole-4^), 7.41 (d, *^4^J*= 1.8 Hz, 1H, 1H, C**H**^indole-7^), 7.27 (d, *^3^J*= 2.4 Hz, 1H, C**H**^indole-2^), 7.02 (dd, *^3^J*= 8.4, *^4^J*= 1.9 Hz, 1H, C**H**^indole‑5^), 4.05 (t, *^3^J*= 6.1 Hz, *^3^J*= 6.1 Hz, 1H, C**H**^α^), 3.23 (dd, *^2^J*= 15.1, *^3^J*= 6.1 Hz, 2H, C**H**_2_^ß^).

#### 7-Bromo-L-tryptophan

**^1^H-NMR** (600 MHz, DMSO-d_6_) [δ/ppm]: 11.28 (s, 1H, N**H**^indole^), 8.34 (s, 2H, N**H_2_**), 7.59 (dd, *^3^J*= 7.9 Hz, *^4^J*= 1.9 Hz, 1H, C**H**^indole-4^), 7.36‑ 7.23 (m, 2H, C**H**^indole-6^, C**H**^indole-2^), 6.97 (ddd, *^3^J*= 7.7 Hz, *^3^J*= 7.7 Hz, *^4^J*= 1.9 Hz, 1H, C**H**^indole-5^), 4.12 (t, *^3^J*= 6.2 Hz, *^3^J*= 6.2 Hz, 1H, C**H**^α^), 3.30 – 3.19 (m, 2H, C**H_2_**^ß^).

**^13^C-NMR** (151 MHz, DMSO-d_6_) [δ/ppm]: 170.8 (1C, **C^q^**O_2_H), 134.5 (1C, **C^q^C**Br), 128.9 (1C, C^γ-indole^**C^q^**), 126.3 (1C, **C^t,^** ^5‑indole^), 123.7 (1C, **C^t,^** ^2-indole^), 120.1 (1C, **C^t,^** ^6^**^-^**^indole^), 117.9 (1C, **C^t,^** ^4^**^-^**^indole^), 108.5 (1C, **C^q,^** ^γ^**^-^**^indole^), 104.2 (1C, **C**Br), 52.7 (1C, **C^α^**), 26.1 (1C, **C^β^**H_2_).

| **ESI-MS** (*m/z*): | Calculated | Determined |
| --- | --- | --- |
|  | [M^79Br^+H]^+^ 283.01 | [M^79Br^+H]^+^ 283.04 |
|  | [M^81Br^+H]^+^ 285.01 | [M^81Br^+H]^+^ 285.04 |

# Enzymatic Synthesis of Tryptophan Derivatives with Tryptophan Synthase

#### Expression and lysis of the cells

The cultivation of the Trp synthase mutant TmAzul was carried out based on the protocol by F. Arnold et al.^[3]^

Precultures of *E.coli* BL21 (DE3) pet28 TmAzul in 10 mL Terrific Broth supplemented with 100 μg/mL kanamycin (TB_kan_) were incubated overnight at 37 °C and 130 rpm. For expression, 5 mL of overnight culture was used to inoculate 750 mL of TB_kan_ in a 2L flask, which was incubated at 37 °C and 130 rpm until OD_600_=0.8 was reached. Cultures were cooled on ice for 30 min. Then overexpression was induced by addition IPTG with a final concentration of 1 mM. Cultures were shaken overnight at 20 °C and 130 rpm. Cells were harvested by centrifugation at 10000 x g for 20 min at 4 °C and stored at −20 °C and washed with KPi-buffer (50 mM KH_2_PO_4_, pH 7.8).

Thawed cell pellets (~14 g) were resuspended in 50 mL (3.6 g/mL) of lysis buffer (100 μM PLP, 50 mM KH_2_PO_4_ buffer, pH 8) and heat treated at 75 °C for 70 min. The lysate was centrifuged for 20 min at 14000 x g and 4 °C and the supernatant collected

#### Gram-Scale Preparation of L-Azuleneylalanine

Azulene (1.0 g, 7.8 mmol) and serine (1.1 eq., 8.6 mmol, 0.9 g) were suspended in DMSO (30 mL, ≤15%) and 50 mM KPi buffer (70 mL). Heat-treated lysate from four 250 mL expression cultures (36 mL) was added and incubated for approximately 48 h under light exclusion (55°C, 130 rpm). The turnover was controlled by analytical HPLC.

The aqueous phase was concentrated under reduced pressure, and residual DMSO was removed by sequential washing with dichloromethane and ethyl acetate. The resulting precipitated amino acid was washed with cold water and ethyl acetate. Azulenenylalanine was subjected to spectroscopic analysis without prior chromatographic desalting and was subsequently employed for Fmoc protection.

#### ß-(1-Azulenyl)-L-alanine

**^1^H-NMR** (600 MHz, DMSO-d_6_) [δ/ppm]: 8.42 (d, ^3^*J* = 9.6 Hz, 1H, C**H^azu-4^**), 8.39 (d, ^3^*J*= 9.4 Hz, 1H, C**H^azu-8^**), 8.33 (s, 3H, N**H_3_**^+^), 7.86 (d, ^3^*J*= 3.8 Hz, 1H, C**H^azu-3^**), 7.70 (t, ^3^*J* = 9.8 Hz, ^3^*J* = 9.8 Hz, 1H, C**H^azu-6^**), 7.39 (d, ^3^*J*= 3.8 Hz, 1H, C**H^azu-2^**), 7.23 (dt, ^3^*J* = 11.5 Hz, ^3^*J* = 9.8 Hz, 2H, C**H^azu-5^**, C**H^azu-7^**), 4.26-4.23 (bs, 1H, C^α^**H**), 3.60 (m, ^3^*J*= 6.5 Hz, ^3^*J*= 6.5 Hz, 2H, C^ß^**H_2_**).

**^13^C-NMR** (151 MHz, DMSO-d_6_) [δ/ppm]: 170.7 (1C, **C**O_2_H), 140.7 (1C, **C^q^**C^azu-4^), 138.2 (1C, **C^azu-6^**), 138.0 (1C**, C^azu-2^**), 136.9 (1C**, C^azu-8^**), 136.7 (1C**, C^azu-4^**), 133.8 (1C**, C^q^**C^azu-8^), 123.1 (1C**, C^azu-5^**), 122.6 (1C, **C^q^**C^ß^H_2_), 122.2 (1C**, C^azu-6^**), 117.1 (1C, **C^azu-3^**), 53.5 (1C, **C**NH_2_), 28.0 (1C, **C**H_2_).

| **HR-MS** (*m/z*) | Calculated | Determined |
| --- | --- | --- |
|  | [M+H]^+^ 216.10191 | [M+H]^+^ 216.1020 |
|  | [M+Na]^+^ 238.08385 | [M+Na]^+^ 238.0839 |

#### Sequence of TmAzul

ATGAAAGGCTACTTCGGTCCGTACGGTGGCCAGTACGTGCCGGAAATCCTGATGGGAGCTCTGGAAGAACTGGAAGCTGCGTACGAAGGAATCATGAAAGATGAGTCTTTCTGGAAAGAATTCAATGACCTGCTGCGCGATTATGCGGGTCGTCCGACTCCGCTGTACTTCGCACGTCGTCTGTCCGAAAAATACGGTGCTCGCGTATATCTGAAACGTGAAGACCTGCTGCATACTGGTGCGCATAAAATCAATAACGCTATCGGCCAGGTTCTGCTGGCAAAACTAATGGGCAAAACCCGTATCATTGCTGAAACGGGTGCTGGTCAGCACGGCGTAGCAACTGCTACCGCAGCAGCGCTGTTCGGTATGGAATGTGTAATCTATATGGGCGAAGAAGACACGATCCGCCAGAAACTAAACGTTGAACGTATGAAACTGCTGGGTGCTAAAGTTGTACCGGTAAAATCCGGTAGCCGTACCCTGAAAGACGCAATTGACGAAGCTCTGCGTGACTGGATTACCAACCTGCAGACCACCTATTACGTGTCCGGCTCTGTGGTTGGTCCGCATCCATATCCGATTATCGTACGTAACTTCCAAAAGGTTATCGGCGAAGAGACCAAAAAACAGATTCCAGAAAAAGAAGGCCGTCTGCCGGACTACATCGTTGCGTGCGTGAGCGGTGGTTCTAACGCTGCCGGTATCTTCTATCCGTTTATCGATTCTGGTGTGAAGCTGATCGGCGTAGAAGCCGGTGGCGAAGGTCTGGAAACCGGTAAACATGCGGCTTCTCTGCTGAAAGGTAAAATCGGCTACCTGCACGGTTCTAAGACGTTCGTTCTGCAGGATGACAGGGGTCAAGTTCAGGTGAGCCACTCCGTCTCCGCTGGCCTGGACTACTCCGGTGTCGGTCCGGAACACGCCTATTGGCGTGAGACCGGTAAAGTGCTGTACGATGCTGTGACCGATGAAGAAGCTCTGGACGCATTCATCGAACTGTCTCGCCTGGAAGGCATCATCCCAGCCCTGGAGTCTTCTCACGCACTGGCTTATCTGAAGAAGATCAACATCAAGGGTAAAGTTGTGGTGGTTAATCTGTCTGGTCGTGGTGACAAGGATCTGGAATCTGTACTGAACCACCCGTATGTTCGCGAACGCATCCGCCTCGAGCACCACCACCACCACCACTGA

# Fmoc protection of tryptophan derivatives

The Fmoc protection of tryptophan was based on known literature.^[4]^

Tryptophan derivatives were dissolved in ACN/H_2_O (20mM), mixed with FmocOSu (1.1eq) and NaHCO_3_ (3eq) and pH 9 was achieved by adding 1 m NaOH solution. The reaction progress was monitored by RP-HPLC. After evaporation of the solvent under reduced pressure, the residue was resuspended in EtOAc and acidified with 4M HCl to a pH-value of 2. After extracting with EtOAc (3×20 mL) the combined organic layers were washed with 5% aq. KHSO_4_, dried over MgSO_4_, and the solvent was evaporated. The crude product was purified by flash column chromatography (0–8% MeOH in DCM/0.1% TFA) and lyophilized to give Fmoc-X-Trp as a colorless solid.

#### Fmoc-5-Bromo-L-tryptophan

^1^H-NMR (600 MHz, DMSO-d_6_) 𝛿 [ppm] = 12.74 (s, 1H, O**H**), 11.08 (s, 1H, N**H**^indole^), 7.87 (d, 1H, ^3^*J* =7.6 Hz, 2H, C**H**^Fmoc 6^, C**H**^Fmoc 6^**^´^**), 7.76 (d, ^4^*J* = 1.9 Hz, 1H, C**H**^indole-4^), 7.72 (d, ^3^*J* = 8.3 Hz, 1H, N**H**), 7.64 (2d, *^3^J*= 7.5 Hz, *^3^J*= 7.5 Hz, 2H, C**H**^Fmoc 3^, C**H**^Fmoc 3´^), 7.40 (dd, ^3^*J* = 7.2 Hz, ^3^*J* = 7.2 Hz, 2H, C**H**^Fmoc 5^, C**H**^Fmoc 5´^), 7.34 – 7.22 (m, 4H, C**H**^Fmoc 4^, C**H**^Fmoc 4´^, C**H**^indole-6^, C**H**^indole-2^), 7.22 – 7.15 (m, 1H, C**H**^indole-7^), 4.24 – 4.14 (m, 4H, OC**H_2_**^Fmoc^, C**H**, C^α^**H**), 3.16 (dd, ^2^*J*= 14.7 Hz, ^3^*J*=4.7 Hz, 1H, C**H**_2_^ß^), 3.00 (dd, ^2^*J*= 14.7 Hz, ^3^*J* = 9.8 Hz, 1H, C**H**_2_^ß^).

| **LC-MS** (*m/z*): | Calculated | Determined |
| --- | --- | --- |
|  | [M^79^+H]^+^ 505.075 | [M^79^+H]^+^ 505.01 |
|  | [M^81^+H]^+^ 507.079 | [M^81^+H]^+^ 507.00 |

#### Fmoc-5-Chloro-L-tryptophan

**^1^H-NMR** (600 MHz, DMSO-d_6_) 𝛿 [ppm] = 12.70 (s, 1H, O**H**), 11.09 (d, ^3^*J*= 6.9 Hz, 1H, N**H**^indole^), 7.88 (2d, ^3^*J*=6.7 Hz, ^3^*J*=6.7 Hz, 2H C**H**^Fmoc 6^, C**H**^Fmoc 6´^), 7.77-7.60 (m, 4H, C**H**^indole-4^, N**H** C**H**^Fmoc 3^, C**H**^Fmoc 3´^), 7.44‑7.23 (m, 6H, C**H**^Fmoc 5^, C**H**^Fmoc 5´^, C**H**^Fmoc 4^, C**H**^Fmoc 4´^, C**H**^indole-6^, C**H**^indole-2^), 7.09-7.04 (m 1H, C**H**^indole-7^), 4.29‑4.16 (m 4H, C**H**^Fmoc 4^, C**H**^Fmoc 4´^, C**H**^indole-6^, C**H**^indole-2^), 3.22- 3.12 (m, 1H, C**H**_2_^ß^), 3.01 (dd, ^2^*J*=14.6 Hz, ^3^*J*= 9.8 Hz, 1H C**H**_2_^ß^).

| **LC-MS** (m/z): | Calculated | Determined |
| --- | --- | --- |
|  | [M+H]^+^ 461.13 | [M+H]^+^ 461.85 |

#### Fmoc-ß-(1-Azulenyl)-L-alanine

Synthesis and analysis are orientated on Moroz *et al.*^[5]^

**^1^H-NMR** (600 MHz, DMSO-d_6_) 𝛿 [ppm] = 12.75 (bs, 1H, O**H**), 8.41 (d, ^3^*J*= 9.7 Hz, 1H, C**H^azu-4^**), 8.33 (d, ^3^*J*= 9.4 Hz, 1H, C**H^azu-8^**), 7.89 (d, ^3^*J* = 7.5 Hz, 2H C**H ^Fmoc-4^**_,_ C**H ^Fmoc-4´^**), 7.87 (d, ^3^*J*= 7.6 Hz, 2H), 7.79 (d, ^3^*J* = 8.4 Hz, 1H, C**H^azu-3^**), 7.69 (d, ^3^*J* = 7.5 Hz, 2H, C**H ^Fmoc-1^**_,_ C**H ^Fmoc-1´^**), 7.68 – 7.58 (m, 2H, N**H,** C**H^azu-6^**), 7.44-7.36 (m, 3H),2d C**H ^Fmoc-3´^**_,_ C**H** **^Fmoc-3,^** C**H^azu-2^**), 7.29 (t, *J* = 7.5 Hz, 1H, C**H^Fmoc-2^**), 7.25 (t, *J* = 7.5 Hz, 1H, C**H^Fmoc-2´^**), 7.18 (dt, ^3^*J* =23.4 Hz, ^3^*J*=9.7 Hz, 2H, C**H^azu-5^**, C**H^azu-7^**), 4.34 – 4.28 (m, 1H, C^α^**H**), 4.26 (d, ^3^*J*= 7.0 Hz, 2H, C**H_2_^Fmoc^**), 4.21 (d, ^3^*J* = 6.8 Hz, 1H, CH_2_C**H^Fmoc^**), 3.56 (dd, ^2^*J* = 14.6 Hz, ^3^*J* =4.8 Hz, 1H, C**H**_2_^ß^), 3.39 (dd, ^2^*J*= 14.6 Hz, ^3^*J=* 10.0 Hz, 1H, C**H**_2_^ß^).

**^13^C-NMR** (151 MHz, DMSO-d_6_) 𝛿 [ppm] = 173.5 (1C, **C**O_2_H), 156.6 (1C, **C**O_2_Fmoc), 144.0 (2C, **C^Fmoc,q^**C^9^H), 140.7 (2C, **C^Fmoc,^** ^q^C^Fmoc-4^H), 140.7 (1C, **C^q^**C^azu-4^), 140.3 (1C**, C^q^**C^azu-8^), 137.8 (1C**, C^azu-2^**), 137.7 (1C**, C^azu-8^**), 136.5 (1C**, C^azu-4^**), 127.6 (2C, **C^Fmoc-3^**H_,_ **C^Fmoc-3´^**H), 127.0 (2C, **C^Fmoc-4^**H_,_ **C^Fmoc-4´^**H), 125.9 (1C**, C^azu-7^**), 125.2 (1C**, C^azu-5^**), 125.1 (2C, **C^Fmoc-2^**H_,_ **C^Fmoc-2´^**H), 122.5 (1C, **C^q^**C^ß^H_2_), 122.0 (1C**, C^azu-6^**), 120.1 (2C, **C^Fmoc-1^**H_,_ **C^Fmoc-1´^**H), 116.7 (1C, **C^azu-3^**), 65.0 (1C, **C^Fmoc^**H_2_), 55.7 (1C, **C^α^**NHFmoc), 46.7 (1C, **C^Fmoc-9^**HCH_2_), 28.7 (1C, **C^ß^**H_2_).

| **HR-MS** (*m/z*) | Calculated | Measured |
| --- | --- | --- |
|  | [M+H]^+^ 438.16999 | [M+H]^+^ 438.1692 |
|  | [M+Na]^+^ 460.15193 | [M+Na]^+^ 460.1507 |

#### Fmoc-6-Bromo-L-tryptophan

**^1^H-NMR** (600 MHz, DMSO-d_6_) 𝛿 [ppm] = 12.70 (s, 1H, O**H**), 11.01 (d, ^3^*J*=2.4 Hz, 1H, N**H**^indole^), 7.88 (d, ^3^*J=*7.6 Hz, 2H, C**H**^Fmoc-6^, C**H**^Fmoc-6´^), 7.70 (d, ^3^*J=* 8.3 Hz, 1H, C**H**^Indol indole-4^), 7.65 (t, ^3^*J=* 8.2 Hz, 2H, C**H**^Fmoc-3^, C**H**^Fmoc-3´^), 7.55-7.51 (m, 2H, N**H**, C**H**^indole7^ ), 7.40 (dd, ^3^*J=* 7.1 Hz, ^3^*J=* 7.1 Hz, 2H, C**H**^Fmoc-5^, C**H**^Fmoc 5´^), 7.35-7.24 (m, 2H, C**H**^Fmoc-4^, C**H**^Fmoc-4´^), 7.21 (d, ^3^*J=* 2.3 Hz, 1H, C**H**^indole2^), 7.11 (dd, ^3^*J=* 8.5 Hz, ^4^*J=*  1.8 Hz, 1H, C**H**^indole-5^), 4.32 – 4.06 (m, 3H, OC**H_2_**^Fmoc^, C**H**_2_^Fmoc^C**H**, C^α^**H**), 3.18 (dd, ^2^*J*= 13.4 Hz, ^3^*J=* 4.5 Hz, 1H, C**H_2_^ß^**), 3.02 (dd, ^2^*J=* 14.6 Hz, ^3^*J=* 9.9 Hz, 1H, C**H**_2_^ß^).

| **ESI-MS** (*m/z*): | Calculated | Determined |
| --- | --- | --- |
|  | [M^79^+H]^+^ 505.075 | [M^79^+H]^+^ 505.09 |
|  | [M^81^+H]^+^ 507.079 | [M^81^+H]^+^ 507.08 |

#### Fmoc-6-Chloro-L-tryptophan

**^1^H-NMR** (600 MHz, DMSO-d_6_) 𝛿 [ppm] = 12.73 (s, 1H, O**H**), 11.01 (d, ^3^*J*=2.4 Hz, 1H, N**H**^indole^), 7.88 (d, ^3^*J*=7.6 Hz, 2H, C**H**^Fmoc-6^, C**H**^Fmoc-6´^), 7.71 (d, ^3^*J* = 8.3 Hz, 1H, C**H**^indole-4^), 7.65 (t, ^3^*J*=8.2 Hz, 2H, C**H**^Fmoc-3^, C**H**^Fmoc-3´^), 7.58 (d, ^3^*J*= 8.4 Hz, 1H , N**H**), 7.44-7.37 (m, 3H, C**H**^indole-7^ ,C**H**^Fmoc-5^, C**H**^Fmoc-5´^), 7.33‑7.25 (m, 2H), 7.22 (d, ^3^*J*= 2.2 Hz, 1H, C**H**^indole-2^), 6.99 (dd, ^3^*J=* 8.4 Hz, ^4^*J=* 1.9 Hz, 1H, C**H**^indole-5^), 4.24-4.14 (m, 4H, OC**H_2_**^Fmoc^, C**H**, C^α^**H**), 3.18 (dd, ^2^*J*= 14.7 Hz, ^3^*J*= 4.5 Hz, 1H, C**H_2_^ß^**), 3.02 (dd, ^2^*J*= 14.6 Hz, ^3^*J*= 9.8 Hz, 1H, C**H_2_^ß^**).

| **ESI-MS** (*m/z*): | Calculated | Determined |
| --- | --- | --- |
|  | [M+H]^+^ 461.13 | [M+H]^+^ 461.14 |

#### Fmoc-7-Bromo-L-tryptophan

**^1^H-NMR** (600 MHz, DMSO-d_6_) 𝛿 [ppm] = 12.69 (s, 1H, O**H**), 11.11 (d, ^3^*J*= 2.6 Hz, 1H, N**H**^indole^), 7.87 (d, ^3^*J*=7.4 Hz, 2H, C**H**^Fmoc-6^, C**H**^Fmoc-6´^), 7.74 (d, ^3^*J*= 8.2 Hz, 1H, N**H**), 7.71-7.54 (m, 3H, CO_2_**H**, C**H**^Fmoc-3^, C**H**^Fmoc-3´^), 7.40 (dd, ^3^*J*=7.4 Hz, 2H, C**H**^Fmoc-5^, C**H**^Fmoc-5´^), 7.33- 7.25 (m, 4H, C**H**^indole-2^, C**H**^indole-6^, C**H**^Fmoc-4^, C**H**^Fmoc-4´^), 6.95 (t, ^3^*J*= 7.7 Hz, 1H, C**H**^indole-5^), 4.26-4.15 (m, 4H, OC**H_2_**^Fmoc^, C**H**, C^α^**H**), 3.20 (dd, ^2^*J*=14.6 Hz, ^3^*J*= 4.6 Hz, 1H, C**H_2_^ß^**), 3.03 (dd, ^2^*J*= 14.6 Hz, ^3^*J*= 9.9 Hz, 1H, C**H_2_^ß^**).

| **ESI-MS (***m/z*): | **Calculated** | **Determined** |
| --- | --- | --- |
|  | [M^79^+H]^+^ 505.075 | [M^79^+H]^+^ 505.06 |
|  | [M^81^+H]^+^ 507.079 | [M^81^+H]^+^ 507.05 |

# Peptide synthesis

Peptides were synthesized manually on *o*-chlorotrityl chloride resin (CTC, maximum loading 1.6 mmol/g) according to the **Fmoc-strategy in a syringe** equipped with a porous polypropylene filter and incubated on an automated shaker. Fmoc deprotections were performed by treating the resin with 20% piperidine in DMF with 0.1 % HOBt (2× 10 min). Sequential washing steps were conducted with DMF.

For **determination of the resin loading**, a sample of the loaded resin (1.00 mg) was incubated in a cleavage solution (3 mL, 20% piperidine in DMF) for 30 min. The absorbance of the resulting solution was measured at λ = 290 nm using 20% piperidine in DMF as a blank. The loading capacity was calculated according to the Lambert–Beer law using the following equation:

$$Resin loading \left[ \frac{\mathrm{mmol}}{g_{loaded resin}} \right]=\frac{A_{290}}{m_{loaded resin}\cdot1.65}$$

**Coupling reactions** of standard Fmoc-protected amino acids (4eq) were carried out using coupling reagents Oxyma (4 eq) and DIC (4 eq) or TBTU and DIPEA for two hours.

Coupling of Fmoc-(XBr)Trp-OH and Fmoc-Lys(N_3_)-OH were performed with less equivalents (1.1 eq) and a longer reaction time (≤15 h) and HOBt and DIPEA instead of Oxyma as coupling reagents.

After every coupling step a **capping** (2x 10 min) was performed by adding acidic anhydride (10eq) and pyridine (10 eq) in DMF.

***N*-Methylation** was carried out according to the protocol of Chatterjee *et al.*^[6]^ Activation of the Fmoc-deprotected amine was achieved by incubating *ortho*-nitrobenzenesulfonyl chloride (4 eq.) and *sym*-Collidine (10 eq.) dissolved in NMP (3x15 min). Methylation (3x) was done with DBU (3 eq, 5min) in NMP, followed by the addition of DMS (10 eq, 10 min). The *o*-NBS-deprotection was carried out by incubating a solution of *ß*-mercaptoethanol (10 eq.) and DBU (5 eq.) in NMP (2x 15 min). Every step was followed by rigorous washing and draining with NMP and DCM. Conversion was determined by test-cleavage and LC-MS-measurements.

For **cleavage,** the resin was incubated with a solution of hexafluoroisopropanol in DCM (1:3, 4x 5 min) and the filtrate was taken up in DCM. After evaporation, the crude peptide was lyophilized.

The **backbone cyclisation** was carried out without prior purification under *pseudo-*dilution-conditions using a syringe pump (0.02 mL/min), with HOBt (1.3 eq), HATU (1.3 eq.) and DIPEA (3 eq.) in DMF (final concentration 0.01 M). After evaporating the solvent, the sidechain deprotection was carried out by stirring the crude peptide in TFA/TIS/H2O (95/2.5/2.5) overnight. After complete cleavage the solution was co-evaporated with toluene and the residue was lyophilized. Peptides were purified with preparative HPLC (0 to 100% H_2_O/0.1%TFA in ACN/0.1%TFA).

# Halogenated Nonapeptides

#### c(DLAFp^(NMe)^K^(5-Br)^WRG) × 2 TFA (**1**)

**^1^H-NMR** (DMSO-d6, 600 MHz) 𝛿 [ppm] = 12.51 (s, 1H, O**H**), 10.97 (d, ^4^*J* = 2.3 Hz, 1H, W-N^1^**H**), 8.73 (d, ^3^*J* = 8.5 Hz, 1H, R-N^α^**H**), 8.53 (d, ^3^*J* = 6.8 Hz, 1H, D-N^α^**H**), 8.00 (d,^4^*J* = 1.9 Hz, 1H, W-C^4^**H**), 7.98 (d, ^3^*J* = 7.0 Hz, 1H, A-N^α^**H**), 7.87 (dd, ^3^*J* = 9.4 Hz, ^3^*J* = 6.8 Hz,1H, G-N^α^**H**), 7.71 – 7.65 (m, 2H, F-N^α^**H**, L-N^α^**H**), 7.63 (t, ^3^*J* = 5.3 Hz, 3H, K-N**H**3^+^), 7.56 – 7.50(m, 1H, R-C=N**H**), 7.43 – 7.39 (m, 1H, W-N^α^**H**), 7.31 (d, ^3^*J* = 8.7 Hz, 1H, W-C^7^**H**), 7.30 – 7.22(m, 4H, F-C**H*^ortho^***, F-C**H*^meta^***), 7.21 – 7.16 (m, 1H, F-C**H*^para^***), 7.16 (dd, ^3^*J* = 8.7 Hz, ^4^*J* = 1.9 Hz,1H, W-C^6^**H**), 7.13 (d, ^4^*J* = 2.3 Hz, 1H, W-C^2^**H**), 6.89 (s, 3H, R-N**H**3^+^), 4.89 - 4.79 (m, 2H,F-C^α^**H**, K-C^α^**H**), 4.81 – 4.73 (m, 1H, W-C^α^**H**), 4.62 (dd, ^3^*J* = 7.9 Hz, ^3^*J* = 5.3 Hz, 1H, p-C^α^**H**),4.48 (dq, ^3^*J* = 7.0 Hz, ^3^*J* = 6.9 Hz, 1H, A-C^α^**H**), 4.42 (ddd, ^3^*J* = 7.5 Hz, ^3^*J* = 7.4 Hz, ^3^*J* = 6.8 Hz,1H, D-C^α^**H**), 4.38 – 4.31 (m, 2H, L-C^α^**H**, R-C^α^**H**), 3.96 (dd, ^2^*J* = 15.6 Hz, ^3^*J* = 5.0 Hz, 1H,G-C^α^**H**2), 3.74 (dd, ^2^*J* = 15.6 Hz, ^3^*J* = 5.4 Hz, 1H, G-C^α^**H**2), 3.39 – 3.31 (m, 1H, p-C^δ^**H**2), 3.20(ddd, ^2^*J* = 9.7 Hz, ^3^*J* = 6.4 Hz, ^3^*J* = 6.4 Hz, 1H, p-C^δ^**H**2), 3.15 – 3.07 (m, 2H, R-C^δ^**H**2), 3.06 – 2.93 (m, 4H, F-C^β^**H**2, W-C^β^**H**2), 2.74 (s, 1H, R-N^ε^**H**), 2.72 – 2.63 (m, 3H, K-C^ε^**H**2, D-C^β^**H**2),2.57 (dd, ^2^*J* = 16.6 Hz, ^3^*J* = 9.3 Hz, 1H, D-C^β^**H**2), 2.42 (s, 3H, K-NC**H**3), 2.09 – 2.01 (m, 1H,p-C^β^**H**2), 2.00 – 1.93 (m, 1H, p-C^β^**H**2), 1.78 – 1.63 (m, 4H, R-C^β^**H**2, L-C^β^**H**2), 1.61 – 1.49 (m,3H, L-C^γ^**H**, L-C^β^**H**), 1.48 – 1.36 (m, 4H, K-C^δ^**H**2, R-C^γ^**H**2), 1.31 – 1.25 (m, 1H, p-C^γ^**H**2), 1.22(d, ^3^*J* = 6.9 Hz, 1H, A-C**H3**), 1.19 – 1.10 (m, 1H, p-C^γ^**H**2), 1.04 (tt, ^3^*J* = 8.0 Hz, ^3^*J* = 7.9 Hz, 2H, K-C^γ^**H**2), 0.84 (dd, ^3^*J* = 15.0 Hz, ^3^*J* = 6.0 Hz, 6H, L-C**H**3).

**^13^C-NMR** (DMSO-d6, 151 MHz) 𝛿 [ppm] = 172.8, 171.9, 171.6, 171.5, 171.4, 170.9,170.4, 169.5, 168.8, 168.7, 156.6, 136.7, 134.8, 129.3, 129.1, 128.2, 126.7, 126.6, 123.2,121.2, 113.3, 111.0, 109.9, 55.9, 55.5, 52.5, 52.2, 51.8, 50.2, 48.4, 46.8, 42.3, 41.7, 40.8,40.3, 38.6, 38.0, 35.4, 29.8, 29.4, 28.2, 26.5, 25.9, 25.2, 24.9, 24.2, 23.3, 22.1, 21.6, 17.7.

| **ESI-MS** (*m/z*): | Calculated  [M^79Br^+H]^+^: 1163.5  [M^81Br^+H]^+^: 1165.5 | Determined  [M^79Br^+H]^+^: 1163.5  [M^81Br^+H]^+^: 1165.5 |
| --- | --- | --- |
| **HR-MS** (*m/z*): | Calculated  [M^79Br^+H]^+^: 1163.5001  [M^81Br^+H]^+^: 1165.4995 | Determined  [M^79Br^+H]^+^: 1163.5033  [M^81Br^+H]^+^: 1165.5031 |

c(DLAFp^(NMe)^K^(6-Br)^WRG) × 2 TFA (**2**)

**^1^H-NMR** (DMSO-d6, 600 MHz) 𝛿 [ppm] = 12.52 (s, 1H, O**H**), 10.93 (s, 1H, W-N^1^**H**), 8.68 (d, ^3^*J* = 7.9 Hz, 1H, R-N^α^**H**), 8.53 (d, ^3^*J* = 6.8 Hz, 1H, D-N^α^**H**), 8.00 (d, ^3^*J* = 7.1 Hz, 1H, A-N^α^**H**), 7.92 – 7.85 (m, 1H, G-N^α^**H**), 7.73 (d, ^3^*J* = 8.2 Hz, 1H, W-C^4^**H**), 7.71 – 7.63 (m, 5H, K-N**H**_3_^+^, L-N^α^**H**, F-N^α^**H**), 7.59 – 7.53 (m, 1H, R-C=N**H**), 7.52 (d, ^4^*J* = 1.7 Hz, 1H, W-C^7^**H**), 7.32 (d, ^3^*J* = 8.9 Hz, 1H, W-N^α^**H**), 7.31 – 7.19 (m, 5H, F-C**H*^ortho^***, F-C**H*^meta^***, F-C**H*^para^***), 7.14 (d, ^3^*J* = 8.2 Hz, 1H, W-C^5^**H**), 7.11 – 7.08 (m, 1H, W-C^2^**H**), 6.95 (s, 3H, R-N**H**_3_^+^), 4.86 (dd, ^3^*J* = 10.9 Hz, ^3^*J* = 4.6 Hz, 1H, K-C^α^**H**), 4.85 – 4.75 (m, 2H, W-C^α^**H**, F-C^α^**H**), 4.57 (dd, ^3^*J* = 7.9 Hz, ^3^*J* = 5.5 Hz, 1H, p-C^α^**H**), 4.45 (dq, ^3^*J* = 7.1 Hz, ^3^*J* = 7.0 Hz, 1H, A-C^α^**H**), 4.42 – 4.32 (m, 2H, L-C^α^**H**, R-C^α^**H**, D-C^α^**H**), 3.93 (dd, ^2^*J* = 15.6 Hz, ^3^*J* = 5.0 Hz, 1H, G-C^α^**H**2), 3.73 (dd, ^2^*J* = 15.6 Hz, ^3^*J* = 5.6 Hz, 1H, G-C^α^**H**2), 3.47 (ddd, ^2^*J* = 10.8 Hz, ^3^*J* = 6.8 Hz, ^3^*J* = 6.8 Hz, 1H, p-C^δ^**H**2), 3.19 (ddd, ^2^*J* = 10.8 Hz, ^3^*J* = 7.0 Hz, ^3^*J* = 7.0 Hz, 1H, p-C^δ^**H**2), 3.13 – 3.03 (m, 3H, W-C^β^**H**2, R-C^δ^**H**2), 3.02 – 2.91 (m, 3H, F-C^β^**H**2, W-C^β^**H**2), 2.74 (s, 1H, R-N^ε^**H**), 2.72 – 2.63 (m, 3H, K-C^ε^**H**2, D-C^β^**H**2), 2.58 (dd, ^2^*J* = 16.8 Hz, ^3^*J* = 9.1 Hz, 1H, D-C^β^**H**2), 2.41 (s, 3H, K-NC**H**3), 2.06 – 1.99 (m, 1H, p-C^γ^**H**2), 1.99 – 1.90 (m, 1H, p-C^γ^**H**2), 1.78 – 1.60 (m, 5H, R-C^β^**H**2, L-C^β^**H**2, p-C^β^**H**2), 1.59 – 1.46 (m, 4H, R-C^β^**H**2, L-C^γ^**H**, L-C^β^**H**), 1.45 – 1.35 (m, 4H, K-C^δ^**H**2, R-C^γ^**H**2), 1.21 (d, ^3^*J* = 7.0 Hz, 1H, A-C**H3**), 1.04 (tt, ^3^*J* = 8.1 Hz, ^3^*J* = 7.9 Hz, 2H, K-C^γ^**H**2), 0.83 (dd, ^3^*J* = 14.8 Hz, ^3^*J* = 6.2 Hz, 6H, L-C**H**3).

**^13^C-NMR** (DMSO-d6, 151 MHz) 𝛿 [ppm] = 173.3, 172.4, 172.1, 172.0, 171.8, 171.4, 170.9, 167.0, 169.3, 169.1, 157.1, 137.5, 137.2, 129.8, 129.6, 128.7, 127.1, 126.7, 123.1, 121.3, 114.3, 114.1, 110.8, 56.4, 56.0, 52.9, 52.7, 52.3, 50.7, 48.9, 47.3, 42.8, 41.2, 40.8, 40.5, 39.1, 38.4, 35.9, 30.3, 29.7, 28.7, 27.0, 25.7, 25.3, 24.7, 23.8, 22.6, 22.0, 21.9, 18.1.

| **ESI-MS** (*m/z*): | Calculated  [M^79Br^+H]^+^: 1163.5  [M^81Br^+H]^+^: 1165.5 | Determined  [M^79Br^+H]^+^: 1163.5  [M^81Br^+H]^+^: 1165.5 |
| --- | --- | --- |
| **HR-MS** (*m/z*): | Calculated  [M^79Br^+H]^+^: 1163.5001  [M^81Br^+H]^+^: 1165.4995 | Determined  [M^79Br^+H]^+^: 1163.4983  [M^81Br^+H]^+^: 1165.4969 |

#### c(DLAFp^(NMe)^K^(7-Br)^WRG) × 2 TFA (**3**)

**^1^H-NMR** (DMSO-d6, 600 MHz) 𝛿 [ppm] = 12.51 (s, 1H, O**H**), 11.00 (s, 1H, W-N^1^**H**),(d, ^3^*J* = 7.5 Hz, 1H, R-N^α^**H**), 8.54 (d, ^3^*J* = 6.8 Hz, 1H, D-N^α^**H**), 8.01 (d, ^3^*J* = 7.1 Hz, 1H,A-N^α^**H**), 7.93 (dd, ^3^*J* = 5.6 Hz, ^3^*J* = 5.4 Hz, 1H, G-N^α^**H**), 7.80 (d, ^3^*J* = 7.8 Hz, 1H, W-C^4^**H**),7.73 (d, ^3^*J* = 7.7 Hz, 1H, W-N^α^**H**), 7.71 – 7.64 (m, 4H, K-N**H**_3_^+^, L-N^α^**H**), 7.60 – 7.54 (m, 1H,R-C=N**H**), 7.37 (d, ^3^*J* = 8.9 Hz, 1H, F-N^α^**H**), 7.31 – 7.19 (m, 5H, W-C^2^**H**, F-C**H*^ortho^***, F-C**H*^meta^***),7.18 – 7.15 (m, 1H, F-C**H*^para^***), 7.14 (d, ^3^*J* = 8.0 Hz, 1H, W-C^6^**H**), 6.98 (s, 3H, R-N**H**_3_^+^), 6.94(dd, ^3^*J* = 8.0 Hz, ^3^*J* = 7.8 Hz, 1H, W-C^5^**H**), 4.87 (dd, ^3^*J* = 10.9 Hz, ^3^*J* = 4.5 Hz, 1H, K-C^α^**H**),4.85 – 4.76 (m, 2H, W-C^α^**H**, F-C^α^**H**), 4.56 (dd, ^3^*J* = 7.9 Hz, ^3^*J* = 5.7 Hz, 1H, p-C^α^**H**), 4.48 (dq,^3^*J* = 7.1 Hz, ^3^*J* = 6.9 Hz, 1H, A-C^α^**H**), 4.41 (ddd, ^3^*J* = 7.5 Hz, ^3^*J* = 7.5 Hz, ^3^*J* = 7.5 Hz, 1H, R-C^α^**H**), 4.39 – 4.31 (m, 2H, L-C^α^**H**, D-C^α^**H**), 3.94 (dd, ^2^*J* = 15.5 Hz, ^3^*J* = 5.4 Hz, 1H, G-C^α^**H**2),3.73 (dd, ^2^*J* = 15.5 Hz, ^3^*J* = 5.6 Hz, 1H, G-C^α^**H**2), 3.45 (ddd, ^2^*J* = 9.8 Hz, ^3^*J* = 7.0 Hz, ^3^*J* = 7.0 Hz, 1H, p-C^δ^**H**2), 3.16 (ddd, ^2^*J* = 9.8 Hz, ^3^*J* = 7.1 Hz, ^3^*J* = 7.1 Hz, 1H, p-C^δ^**H**2), 3.13 – 3.04 (m, 4H, W-C^β^**H**2, R-C^δ^**H**2), 2.99 (dd, ^2^*J* = 13.3 Hz, ^3^*J* = 7.7 Hz, 1H, F-C^β^**H**2), 2.94 (dd,^2^*J* = 13.3 Hz, ^3^*J* = 5.5 Hz, 1H, F-C^β^**H**2), 2.76 (s, 1H, R-N^ε^**H**), 2.72 – 2.62 (m, 3H, K-C^ε^**H**2,D-C^β^**H**2), 2.58 (dd, ^2^*J* = 16.6 Hz, ^3^*J* = 9.3 Hz, 1H, D-C^β^**H**2), 2.44 (s, 3H, K-NC**H**3), 2.07 – 1.98(m, 1H, p-C^γ^**H**2), 1.99 – 1.90 (m, 1H, p-C^γ^**H**2), 1.79 – 1.58 (m, 5H, R-C^β^**H**2, L-C^β^**H**2, p-C^β^**H**2),1.59 – 1.38 (m, 8H, R-C^β^**H**2, R-C^γ^**H**2, K-C^δ^**H**2, L-C^γ^**H**, L-C^β^**H**), 1.21 (d, ^3^*J* = 6.9 Hz, 1H, A-C**H3**),1.05 (tt, ^3^*J* = 8.4 Hz, ^3^*J* = 8.0 Hz, 2H, K-C^γ^**H**2), 0.85 (dd, ^3^*J* = 14.0 Hz, ^3^*J* = 6.2 Hz, 6H, L-C**H**3).

**^13^C-NMR** (DMSO-d6, 151 MHz) 𝛿 [ppm] = 172.8, 172.0, 171.6, 171.5, 171.4, 170.4,170.2, 169.5, 168.9, 168.7, 156.6, 136.7, 134.4, 129.3, 129.1, 128.3, 126.6, 126.0, 122.7,119.9, 116.0, 111.6, 104.0, 55.9, 55.6, 52.5, 52.3, 51.8, 50.3, 48.4, 46.8, 42.4, 40.8, 40.3,40.1, 38.6, 38.1, 35.4, 29.9, 29.3, 28.2, 26.5, 25.9, 25.2, 24.8, 24.2, 23.3, 22.1, 21.6, 17.6.

| **ESI-MS** (*m/z*): | Calculated  [M^79Br^+H]^+^: 1163.5  [M^81Br^+H]^+^: 1165.5 | Determined  [M^79Br^+H]^+^: 1163.5  [M^81Br^+H]^+^: 1165.5 |
| --- | --- | --- |
| **HR-MS** (*m/z*): | Calculated  [M^79Br^+H]^+^: 1163.5001  [M^81Br^+H]^+^: 1165.4995 | Determined  [M^79Br^+H]^+^: 1163.5006  [M^81Br^+H]^+^: 1165.5002 |

#### c(DLAWp^(NMe)^K^(6-Br)^WRG) × 2 TFA (**4**)

**^1^H-NMR** (DMSO-d6, 600 MHz) 𝛿 [ppm] = 12.51 (s, 1H, O**H**), 11.93 (d, ^4^*J* = 2.4 Hz, 1H, W^KR^-N^1^**H**), 10.91 (d, ^4^*J* = 2.4 Hz, 1H, W^pA^-N^1^**H**), 8.72 (s, 1H, R-N^α^**H**), 8.57 (d, ^3^*J* = 6.7 Hz, 1H,D-N^α^**H**), 8.07 (d, ^3^*J* = 7.1 Hz, 1H, A-N^α^**H**), 7.86 (s, 1H, G-N^α^**H**), 7.80 (d, ^3^*J* = 7.9 Hz, 1H,W^pA^-N^α^**H**), 7.76 (d, ^3^*J* = 8.3 Hz, 1H, W^KR^-C^4^**H**), 7.66 (d, ^3^*J* = 9.0 Hz, 1H, L-N^α^**H**), 7.63 (t,^3^*J* = 5.9 Hz, ^3^*J* = 5.5 Hz, 3H, K-N**H_3_**^+^), 7.56 (d, ^3^*J* = 7.8 Hz, 1H, W^pA^-C^4^**H**), 7.55 – 7.53 (m, 1H,R-C=N**H**), 7.54 (d, ^4^*J* = 1.8 Hz, 1H, W^KR^-C^7^**H**), 7.43 (d, ^3^*J* = 9.0 Hz, 1H, W^KR^-N^α^**H**), 7.36 (d,^3^*J* = 8.1 Hz, 1H, W^KR^-C^7^**H**), 7.23 (d, ^4^*J* = 2.4 Hz, 1H, W^KR^-C^2^**H**), 7.15 (d, ^4^*J* = 2.4 Hz, 1H,W^pA^-C^2^**H**), 7.12 – 7.07 (m, 2H, W^KR^-C^5^**H**, W^pA^-C^6^**H**), 6.99 (t, ^3^*J* = 7.8 Hz, ^4^*J* = 7.4 Hz, 1H, W^pA^-C^5^**H**), 6.88 (s, 3H, R-N**H_3_^+^**), 4.91 – 4.82 (m, 2H, W^KR^-C^α^**H**, p-C^α^**H**), 4.81 (td, ^3^*J* = 7.9 Hz,^3^*J* = 4.8 Hz, 1H, W^pA^-C^α^**H**), 4.53 (dq, ^3^*J* = 7.1 Hz, ^3^*J* = 6.9 Hz, 1H, A-C^α^**H**), 4.46 (ddd,^3^*J* = 7.6 Hz, ^3^*J* = 7.5 Hz, ^3^*J* = 7.5 Hz, 1H, R-C^α^**H**), 4.39 – 4.29 (m, 3H, L-C^α^**H**, D-C^α^**H**, K-C^α^**H**),3.93 (dd, ^2^*J* = 15.4 Hz, ^3^*J* = 4.7 Hz, 1H, G-C^α^**H**_2_), 3.77 (dd, ^2^*J* = 15.4 Hz, ^3^*J* = 5.7 Hz, 1H, G-C^α^**H**_2_), 3.29 – 3.24 (m, p-C^δ^**H**_2_), 3.18 – 3.04 (m, 6H, R-C^δ^**H**_2_, W^KR^-C^β^**H**_2_, W^pA^-C^β^**H**_2_), 2.78 – 2.70 (m, 1H, p-C^δ^**H**_2_), 2.70 – 2.64 (m, 3H, K-C^ε^**H**_2_, D-C^β^**H**_2_), 2.58 (dd, ^2^*J* = 16.7 Hz,^3^*J* = 9.5 Hz, 1H, D-C^β^**H**_2_), 2.53 – 2.51 (m, 1H, R-N^ε^**H**), 2.40 (s, 3H, K-NC**H**_3_), 1.80 – 1.70 (m,4H, K-C^β^**H**_2_, p-C^β^**H**_2_, R-C^β^**H**_2_), 1.60 – 1.48 (m, 5H, R-C^β^**H**_2_, p-C^γ^**H**_2_, L-C^β^**H**_2_, L-C^γ^**H**), 1.47 – 1.37 (m, 4H, K-C^δ^**H**_2_, R-C^γ^**H**_2_), 1.29 – 1.22 (m, 1H, p-C^γ^**H**_2_), 1.25 (d, ^3^*J* = 6.9 Hz, 1H, A-C**H_3_**),1.18 – 1.12 (m, 1H, p-C^γ^**H**_2_), 1.07 – 0.98 (m, 2H, K-C^γ^**H**_2_), 0.84 (dd, ^3^*J* = 12.6 Hz, ^3^*J* = 5.8 Hz,6H, L-C**H**_3_).

**^13^C-NMR** (DMSO-d6, 151 MHz) 𝛿 [ppm] = 172.9, 172.1, 172.0, 171.6, 171.5, 170.9,170.4, 169.5, 169.4, 168.8, 156.6, 137.0, 136.1, 127.3, 126.3, 125.6, 123.8, 121.1, 120.9,120.8, 118.3, 113.8, 113.7, 111.5, 110.5, 109.2, 99.5, 55.8, 55.6, 52.4, 52.1, 51.7, 50.2, 48.4,46.7, 42.4, 40.8, 40.3, 40.0, 38.9, 35.4, 29.9, 29.4, 28.8, 28.1, 26.5, 26.0, 25.2, 24.6, 24.2,23.3, 22.1, 21.7, 17.6.

| **ESI-MS** (*m/z*): | Calculated  [M^79Br^+H]^+^: 1202.5  [M^81Br^+H]^+^: 1204.5 | Determined  [M^79Br^+H]^+^: 1202.5  [M^81Br^+H]^+^: 1204.5 |
| --- | --- | --- |
| **HR-MS** (*m/z*): | Calculated  [M^79Br^+H]^+^: 1202.5267  [M^81Br^+H]^+^: 1204.5261 | Determined  [M^79Br^+H]^+^: 1202.5241  [M^81Br^+H]^+^: 1204.5170 |

#### c(DLA^(5-Cl)^Wp^(NMe)^KFRG) × 2 TFA (**5**)

| **ESI-MS** (m/z): | Calculated | Determined |
| --- | --- | --- |
|  | [M+H]^+^ 1119.550 | [M+H]^+^ 1119.56 |

#### c(DLA^(6-Cl)^Wp^(NMe)^KFRG) × 2 TFA (**6**)

**^1^H-NMR** (600 MHz, DMSO-d_6_) 𝛿 [ppm] = 12.54 (s, 1H, O**H^Asp^**), 11.10 (d, ^3^*J* =2.4 Hz, 1H, N**H^Indole^**), 8.63 (d, ^3^*J* = 6.0 Hz, 2H, N**H^Arg^**, N**H^Asp^**), 7.90 (d, ^3^*J* = 6.4 Hz, 2H, N**H^Ala^**, N**H^Gly^**), 7.73 – 7.62 (m, 3H, N**H^Trp^**, N**H^Leu^**, C**H^Trp-4^**), 7.54 (d, ^3^*J* = 8.4 Hz, 1H, C^Arg^=N**H**), 7.42 (d, ^3^*J*= 9.2 Hz, 1H, N**H^Phe^**), 7.37 (d, ^4^*J* = 1.9 Hz, 1H, C**H^Trp-2^**) , 7.32 (d, ^4^*J* = 2.5 Hz, 1H, C**H^Trp-7^**), 7.30 – 7.17 (m, 5H, C**H^Phe-^*^ortho^***, C**H^Phe-^*^meta^***, C**H^Phe-^*^para^***, C**H^Trp-5^** , C**H^Trp-2^**), 7.01 (dd, ^3^*J* = 8.4, 1.9 Hz, 2H, C^Arg^N**H_3_^+^**), 4.87-4.76 (m, 3H, C^α^**H^Trp^**, C^α^**H^Phe^**, C^α^**H^pro^**), 4.49- 4.39 (m 2H, C^α^**H^Ala^**, C^α^**H^Arg^**), 4.37 – 4.28 (m, 3H, C^α^**H^Lys^_,_** C^α^**H^Leu^_,_** C^α^**H^Asp^**), 4.03 (dd, ^2^*J*= 15.6 Hz, ^3^*J*= 5.3 Hz, 1H, C^α^**H**_2_**^Gly^**), 3.71 (dd, ^2^*J*= 15.6 Hz, ^3^*J*= 5.1 Hz, 1H, C^α^**H**_2_**^Gly^**), 3.39 (dt, ^2^*J*= 10.0 Hz, *^3^J=*7.0 Hz, 1H, C^δ^**H**_2_**^pro^**), 3.16-3.03 (m, 5H, C^δ^**H_2_^Arg^**, C^ß^**H**_2_**^Phe^**, C^ß^**H_2_^Trp^**), 3.03-2.99 (m, 1H, C^δ^**H**_2_**^pro^**), 2.86 – 2.79 (m, 1H, C**H**_2_**^Phe^**), 2.74 – 2.62 (m, 3H, C^ε^**H_2_^Lys^**, C^ß^**H**_2_**^Asp^**), 2.57 (dd, ^2^*J* = 16.7 Hz, ^3^*J*=9.5 Hz, 1H, C^ß^**H**_2_**^Asp^**), 2.34 (s, 3H, C**H_3_^Lys^**), 1.94-1.84 (m, 2H, C^ß^**H**_2_**^pro^**, C^ß^**H_2_^Lys^**), 1.84- 1.75 (m, 1H, C^ß^**H**_2_**^Lys^**), 1.70 (p, ^3^*J* = 6.9 Hz, 1H, C^ß^**H_2_^Arg^**), 1.63 (td, ^2^*J* = 9.0 Hz, ^3^J=4.7 Hz, 1H, C^ß^**H_2_^pro^**), 1.58 – 1.51 (m, 3H, C^ß^**H_2_^Leu^**, C^ß^**H_2_^Arg^**), 1.50–1.39 (m, 6H, C^γ^**H**_2_**^pro^**, C^γ^**H^Leu^**, C^δ^**H_2_^Lys^**, C^γ^**H_2_^Arg^**), 1.26 (d, ^3^*J* = 6.9 Hz, 3H, C**H_3_^Ala^**). 1.25 – 1.17 (m, 1H, C^γ^**H**_2_**^pro^**), 1.09-1.00 (m, 2H, CH_2_**^Lys-γ^**), 0.84 (dd, ^2^*J* = 10.3 Hz, ^3^*J*= 5.8 Hz, 6H, C**H_3_^Leu^**).

**^13^C-NMR** (126 MHz, DMSO-d_6_) [δ/ppm] = 172.9, 171.6, 171.5, 171.5, 171.4, 170.8, 170.5, 169.7, 169.1, 168.9, 156.7, 137.9, 136.5, 129.4, 127.9, 126.3, 126.2, 125.7, 125.3, 119.4, 118.6, 111.2, 109.34, 55.80, 55.6, 53.2, 52.0, 51.5, 50.2, 48.6, 46.8, 42.2, 35.4, 30.0, 29.6, 28.2, 27.9, 26.5, 26.0, 25.23, 24.9, 24.2, 23.3, 22.1, 21.7, 17.8.

| **HRMS** (m/z): | **Calculated** | **Determined** |
| --- | --- | --- |
|  | [M+H]^+^ 1119.55010 | [M+H]^+^ 1119.5493 |
|  | [M+2H]^2+^ 560.27869 | [M+2H]^2+^ 560.2794 |

#### c(DLA^(5-Br)^Wp^(NMe)^KFRG) × 2 TFA (**7**)

**^1^H-NMR** (DMSO-d6, 600 MHz) 𝛿 [ppm] = 12.51 (s, 1H, O**H**), 11.20 (s, 1H, W-N^1^**H**), 8.67 (d, ^3^*J* = 8.6 Hz, 1H, R-N^α^**H**), 8.64 (d, ^3^*J* = 6.5 Hz, 1H, D-N^α^**H**), 7.94 (d, ^3^*J* = 6.9 Hz, 1H, A-N^α^**H**), 7.89 (dd, ^3^*J* = 5.3 Hz, ^3^*J* = 5.2 Hz, 1H, G-N^α^**H**), 7.70 (d, ^3^*J* = 7.5 Hz, 1H, W-N^α^**H**), 7.68 – 7.60 (m, 5H, L-N^α^**H**, W-C^4^**H**, K-N**H_3_^+^**), 7.58 – 7.53 (m, 1H, R-C=N**H**), 7.47 (d, ^3^*J* = 9.2 Hz, 1H, F-N^α^**H**), 7.37 (d, ^4^*J* = 2.0 Hz, 1H, W-C^2^**H**), 7.34 (d, ^3^*J* = 8.6 Hz, 1H, W-C^7^**H**), 7.28 – 7.24 (m, 4H, F-C**H*^ortho^***, F-C**H*^meta^***), 7.23 (dd, ^3^*J* = 8.6 Hz, ^4^*J* = 2.0 Hz, 1H, W-C^6^**H**), 7.21 – 7.17 (m, 1H, F-C**H*^para^***), 6.92 (s, 3H, R-N**H_3_^+^**), 4.90 – 4.82 (m, 2H, F-C^α^**H**, p-C^α^**H**), 4.72 (ddd, ^3^*J* = 7.5 Hz, ^3^*J* = 7.6 Hz, ^3^*J* = 4.1 Hz, 1H, W-C^α^**H**), 4.51 – 4.41 (m, 2H, A-C^α^**H**, R-C^α^**H**), 4.36 – 4.27 (m, 3H, K-C^α^**H**, L-C^α^**H**, D-C^α^**H**), 4.02 (dd, ^2^*J* = 15.4 Hz, ^3^*J* = 5.3 Hz, 1H, G-C^α^**H**_2_), 3.72 (dd, ^2^*J* = 15.4 Hz, ^3^*J* = 5.2 Hz, 1H, G-C^α^**H**_2_), 3.36 – 3.31 (m, 1H, p-C^δ^**H**_2_), 3.17 (dd, ^2^*J* = 13.9 Hz, ^3^*J* = 4.1 Hz, 1H, W-C^β^**H**_2_), 3.14 – 3.00 (m, 4H, W-C^δ^**H**_2_, F-C^β^**H**_2_, R-C^δ^**H**_2_), 2.89 (t, ^2^*J* = 12.7 Hz, 1H, F-C^β^**H**_2_), 2.84 – 2.78 (m, 1H, p-C^δ^**H**_2_), 2.71 – 2.62 (m, 3H, K-C^ε^**H**_2_, D-C^β^**H**_2_), 2.56 (dd, ^2^*J* = 16.7 Hz, ^3^*J* = 9.7 Hz, 1H, D-C^β^**H**_2_), 2.52 – 2.46 (m, 1H, R-N^ε^**H**), 2.43 (s, 3H, K-NC**H**_3_), 1.85 – 1.75 (m, 3H, K-C^β^**H**2, p-C^β^**H**_2_), 1.75 – 1.68 (m, 1H, R-C^β^**H**_2_), 1.66 – 1.58 (m, 1H, p-C^β^**H**_2_), 1.57 – 1.47 (m, 4H, L-C^β^**H**_2_, L-C^γ^**H**, R-C^β^**H**_2_), 1.46 – 1.36 (m, 4H, K-C^δ^**H**_2_, R-C^γ^**H**_2_), 1.27 (d, ^3^*J* = 6.9 Hz, 1H, A-C**H**_3_), 1.25 – 1.20 (m, 1H, p-C^γ^**H**_2_), 1.18 – 1.11 (m, 1H, p-C^γ^**H**_2_), 1.10 – 0.98 (m, 2H, K-C^γ^**H**_2_), 0.84 (dd, ^3^*J* = 11.4 Hz, ^3^*J* = 5.8 Hz, 6H, L-C**H**_3_).

**^13^C-NMR** (DMSO-d6, 151 MHz) 𝛿 [ppm] = 172.8, 171.6, 171.5, 171.4, 171.3, 170.7, 170.4, 169.7, 169.4, 168.8, 156.6, 137.8, 134.8, 129.4, 129.1, 128.0, 126.0, 123.4, 122.7, 119.9, 113.6, 111.0, 108.8, 55.8, 55.6, 53.3, 52.1, 51.9, 50.1, 48.5, 46.7, 42.2, 40.7, 40.3, 40.1, 38.6, 38.2, 35.4, 30.3, 29.7, 28.1, 26.5, 26.1, 25.2, 24.7, 24.2, 23.3, 22.1, 21.7, 17.8.

| **ESI-MS** (*m/z*): | Calculated  [M^79Br^+H]^+^: 1163.5  [M^81Br^+H]^+^: 1165.5 | Determined  [M^79Br^+H]^+^: 1163.5  [M^81Br^+H]^+^: 1165.5 |
| --- | --- | --- |
| **HR-MS** (*m/z*): | Calculated  [M^79Br^+H]^+^: 1163.5001  [M^81Br^+H]^+^: 1165.4995 | Determined  [M^79Br^+H]^+^: 1163.5023 [M^81Br^+H]^+^: 1165.4996 |

#### c(DLA^(6-Br)^Wp^(NMe)^KFRG) × 2 TFA (**8**)

**^1^H-NMR** (DMSO-d6, 600 MHz) 𝛿 [ppm] = 12.28 (s, 1H, O**H**), 10.95 (s, 1H, W-N^1^**H**), 8.50 (d, ^3^*J* = 8.1 Hz, 1H, R-N^α^**H**), 8.46 (d, ^3^*J* = 7.0 Hz, 1H, D-N^α^**H**), 8.06 (d, ^3^*J* = 7.4 Hz, 1H, A-N^α^**H**), 7.71 (dd, ^3^*J* = 5.9 Hz, ^3^*J* = 4.5 Hz, 1H, G-N^α^**H**), 7.67 (d, ^3^*J* = 7.5 Hz, 1H, W-N^α^**H**), 7.61 (s, 3H, K-N**H_3_^+^**), 7.52 (m, 2H, W-C^4^**H**, L-N^α^**H**), 7.48 (m, 2H, W-C^2^**H**, R-C=N**H**), 7.41 (d, ^3^*J* = 9.1 Hz, 1H, F-N^α^**H**), 7.27 – 7.23 (m, 4H, F-C**H*^ortho^***, F-C**H*^meta^***), 7.23 (d, ^4^*J* = 2.3 Hz, 1H, W-C^7^**H**), 7.20 (dd, ^3^*J* = 5.5 Hz, ^3^*J* = 5.7 Hz, 1H, F-C**H*^para^***), 7.12 (dd, ^3^*J* = 8.4 Hz, ^4^*J* = 1.8 Hz, 1H, W-C^5^**H**), 6.99 (s, 3H, R-N**H_3_^+^**), 4.91 – 4.80 (m, 3H, W-C^α^**H**, F-C^α^**H**, p-C^α^**H**), 4.59 (dq, ^3^*J* = 7.4 Hz, ^3^*J* = 7.0 Hz, 1H, A-C^α^**H**), 4.44 (ddd, ^3^*J* = 8.1 Hz, ^3^*J* = 7.5 Hz, ^3^*J* = 7.5 Hz, 1H, R-C^α^**H**), 4.41 – 4.34 (m, 3H, L-C^α^**H**, D-C^α^**H**, K-C^α^**H**), 3.89 (dd, ^2^*J* = 15.3 Hz, ^3^*J* = 4.5 Hz, 1H, G-C^α^**H**_2_), 3.80 (dd, ^2^*J* = 15.3 Hz, ^3^*J* = 5.9 Hz, 1H, G-C^α^**H**_2_), 3.37 (ddd, ^2^*J* = 13.6 Hz, ^3^*J* = 6.8 Hz, ^3^*J* = 6.8 Hz, 1H, p-C^δ^**H**_2_), 3.16 – 3.04 (m, 5H, W-C^β^**H**_2_, F-C^β^**H**2, R-C^δ^**H**_2_), 3.02 – 2.95 (m, 1H, p-C^δ^**H**_2_), 2.95 – 2.87 (m, 1H, F-C^β^**H**_2_), 2.75 – 2.66 (m, 3H, K-C^ε^**H**2, D-C^β^**H**_2_), 2.60 (dd, ^2^*J* = 16.5 Hz, ^3^*J* = 7.0 Hz, 1H, D-C^β^**H**_2_), 2.51 – 2.47 (m, 1H, R-N^ε^**H**), 2.43 (s, 3H, K-NC**H**_3_), 1.94 – 1.86 (m, 2H, K-C^β^**H**_2_, p-C^β^**H**_2_), 1.85 – 1.78 (m, 1H, K-C^β^**H**_2_), 1.77 – 1.70 (m, 1H, R-C^β^**H**_2_), 1.68 – 1.61 (m, 1H, p-C^β^**H**_2_), 1.60 – 1.51 (m, 3H, L-C^β^**H**_2_, R-C^β^**H**_2_), 1.50 – 1.43 (m, 5H, L-C^γ^**H**, K-C^δ^**H**_2_, R-C^γ^**H**_2_), 1.44 – 1.37 (m, 1H, p-C^γ^**H**_2_), 1.33 – 1.23 (m, 1H, p-C^γ^**H**_2_), 1.27 (d, ^3^*J* = 7.0 Hz, 1H, A-C**H**_3_), 1.10 (tt, ^3^*J* = 7.5 Hz, ^3^*J* = 6.4 Hz, 2H, K-C^γ^**H**_2_), 0.85 (dd, ^3^*J* = 12.6 Hz, ^3^*J* = 6.2 Hz, 6H, L-C**H**_3_).

**^13^C-NMR** (DMSO-d6, 151 MHz) 𝛿 [ppm] = 172.6, 171.5, 171.4, 171.2, 171.0, 170.5, 170.0, 169.1, 168.9, 168.6, 156.6, 137.6, 136.8, 129.1, 127.6, 126.2, 125.9, 124.7, 120.9, 119.6, 113.9, 113.6, 109.4, 55.6, 55.5, 53.0, 52.0, 51.3, 50.2, 48.2, 46.6, 42.3, 40.9, 40.2, 40.1, 38.5, 37.8, 35.3, 29.7, 29.2, 27.9, 26.3, 25.9, 24.9, 24.5, 24.0, 22.9, 22.0, 21.6, 17.4.

| **ESI-MS** (*m/z*): | Calculated  [M^79Br^+H]^+^: 1163.5  [M^81Br^+H]^+^: 1165.5 | Determined  [M^79Br^+H]^+^: 1163.5 [M^81Br^+H]^+^: 1165.5 |
| --- | --- | --- |
| **HR-MS** (*m/z*): | Calculated  [M^79Br^+H]^+^: 1163.5001  [M^81Br^+H]^+^: 1165.4995 | Determined  [M^79Br^+H]^+^: 1163.5026 [M^81Br^+H]^+^: 1165.5026 |

#### c(DLAW^(7-Br)^p^(NMe)^KFRG) × 2 TFA (**9**)

**^1^H-NMR** (DMSO-d6, 600 MHz) 𝛿 [ppm] = 12.28 (s, 1H, O**H**), 11.01 (s, 1H, W-N^1^**H**),8.50 (d, ^3^*J* = 8.0 Hz, 1H, R-N^α^**H**), 8.45 (d, ^3^*J* = 7.1 Hz, 1H, D-N^α^**H**), 8.04 (d, ^3^*J* = 7.4 Hz, 1H,A-N^α^**H**), 7.72 (dd, ^3^*J* = 4.5 Hz, ^3^*J* = 5.7 Hz, 1H, G-N^α^**H**), 7.68 (d, ^3^*J* = 7.6 Hz, 1H, W-N^α^**H**),7.63 (s, 3H, K-N**H_3_^+^**), 7.60 (d, ^3^*J* = 7.8 Hz, 1H, W-C^4^**H**), 7.50 (d, ^3^*J* = 8.9 Hz, 1H, L-N^α^**H**), 7.49(s, 1H, R-C=N**H**), 7.40 (d, ^3^*J* = 9.0 Hz, 1H, F-N^α^**H**), 7.31 (d, ^3^*J* = 7.6 Hz, 1H, W-C^6^**H**), 7.30 – 7.23 (m, 5H, W-C^2^**H**, F-C**H*^ortho^***, F-C**H*^meta^***), 7.20 (dd, ^3^*J* = 7.1 Hz, ^3^*J* = 6.8 Hz, 1H, F-C**H*^para^***),7.01 (s, 3H, R-N**H_3_^+^**), 6.96 (dd, ^3^*J* = 7.8 Hz, ^3^*J* = 7.6 Hz, 1H, W-C^5^**H**), 4.93 – 4.79 (m, 3H,W-C^α^**H**, F-C^α^**H**, p-C^α^**H**), 4.57 (dq, ^3^*J* = 7.4 Hz, ^3^*J* = 6.7 Hz, 1H, A-C^α^**H**), 4.45 (ddd, ^3^*J* = 8.0 Hz,^3^*J* = 7.5 Hz, ^3^*J* = 7.5 Hz, 1H, R-C^α^**H**), 4.41 – 4.34 (m, 2H, L-C^α^**H**, D-C^α^**H**), 4.33 (t, ^3^*J* = 6.6 Hz,1H, K-C^α^**H**), 3.89 (dd, ^2^*J* = 15.4 Hz, ^3^*J* = 4.5 Hz, 1H, G-C^α^**H**2), 3.80 (dd, ^2^*J* = 15.4 Hz,^3^*J* = 5.7 Hz, 1H, G-C^α^**H**2), 3.41 – 3.33 (m, 1H, p-C^δ^**H**2), 3.15 – 3.07 (m, 5H, W-C^β^**H**2, F-C^β^**H**2,R-C^δ^**H**2), 3.03 – 2.96 (m, 2H, p-C^δ^**H**2, F-C^β^**H**2), 2.73 – 2.65 (m, 3H, K-C^ε^**H**2, D-C^β^**H**2), 2.60 (dd,^2^*J* = 16.7 Hz, ^3^*J* = 9.2 Hz, 1H, D-C^β^**H**2), 2.52 – 2.46 (m, 1H, R-N^ε^**H**), 2.44 (d, ^4^*J* = 9.2 Hz, 3H,K-NC**H**3), 1.91 – 1.79 (m, 3H, K-C^β^**H**2, p-C^β^**H**2), 1.79 – 1.71 (m, 1H, R-C^β^**H**2), 1.67 – 1.60 (m,1H, p-C^β^**H**2), 1.59 – 1.52 (m, 3H, L-C^β^**H**2, R-C^β^**H**2), 1.50 – 1.42 (m, 5H, L-C^γ^**H**, K-C^δ^**H**2,R-C^γ^**H**2), 1.35 (m, 1H, p-C^γ^**H**2), 1.32 – 1.24 (m, 1H, p-C^γ^**H**2), 1.27 (d, ^3^*J* = 6.7 Hz, 1H, A-C**H3**),1.10 (tt, ^3^*J* = 7.6 Hz, ^3^*J* = 7.5 Hz, 2H, K-C^γ^**H**2), 0.85 (dd, ^3^*J* = 12.5 Hz, ^3^*J* = 6.4 Hz, 6H, L-C**H**3).

**^13^C-NMR** (DMSO-d6, 151 MHz) 𝛿 [ppm] = 172.9, 171.6, 171.5, 171.4, 171.3, 170.8,170.5, 169.6, 169.1, 168.9, 156.6, 137.9, 134.4, 129.3, 129.1, 127.9, 126.2, 125.4, 123.7,119.7, 117.7, 110.5, 104.3, 55.7, 55.6, 53.1, 52.0, 51.4, 50.2, 48.6, 46.8, 42.2, 40.7, 0.3,40.1, 38.6, 38.0, 35.4, 29.9, 29.6, 28.2, 26.5, 26.0, 25.2, 24.8, 24.2, 23.3, 22.1, 21.7, 17.8.

| **ESI-MS** (*m/z*): | Calculated  [M^79Br^+H]^+^: 1163.5  [M^81Br^+H]^+^: 1165.5 | Determined [M^79Br^+H]^+^: 1163.5  [M^81Br^+H]^+^: 1165.5 |
| --- | --- | --- |
| **HR-MS** (*m/z*): | Calculated  [M^79Br^+H]^+^: 1163.5001  [M^81Br^+H]^+^: 1165.4995 | Determined  [M^79Br^+H]^+^: 1163.5004  [M^81Br^+H]^+^: 1165.4983 |

#### c(*DLA^(6-Br)^Wp^(NMe)^KWRG) × 2 TFA (****10****)*

**^1^H-NMR** (DMSO-d6, 600 MHz) 𝛿 [ppm] = 12.51 (s, 1H, O**H**), 11.09 (d, ^4^*J* = 2.4 Hz, 1H, W^pA^-N^1^**H**), 10.77 (d, ^4^*J* = 2.3 Hz, 1H, W^KR^-N^1^**H**), 8.70 (d, ^3^*J* = 7.8 Hz, 1H, R-N^α^**H**), 8.56 (d,^3^*J* = 6.8 Hz, 1H, D-N^α^**H**), 8.08 (d, ^3^*J* = 7.2 Hz, 1H, A-N^α^**H**), 7.86 (d, ^3^*J* = 7.8 Hz, 1H, W^pA^-N^α^**H**),7.81 (dd, ^3^*J* = 5.6 Hz, ^3^*J* = 5.2 Hz, 1H, G-N^α^**H**), 7.77 (d, ^3^*J* = 7.6 Hz, 1H, W^KR^-C^7^**H**), 7.67 – 7.62 (m, 4H, L-N^α^**H**, K-N**H**_3_^+^), 7.57 – 7.54 (m, 1H, R-C=N**H**), 7.53 (d, ^4^*J* = 1.8 Hz, 1H, W^pA^-C^7^**H**), 7.52 (d, ^3^*J* = 8.5 Hz, 1H, W^pA^-C^4^**H**), 7.44 (d, ^3^*J* = 8.9 Hz, 1H, W^KR^-N^α^**H**), 7.35 (d,^3^*J* = 8.1 Hz, 1H, W^KR^-C^4^**H**), 7.25 (d, ^4^*J* = 2.4 Hz, 1H, W^pA^-C^2^**H**), 7.21 (d, ^4^*J* = 2.3 Hz, 1H,W^KR^-C^2^**H**), 7.09 (dd, ^3^*J* = 8.5 Hz, ^4^*J* = 1.8 Hz, 1H, W^pA^-C^5^**H**), 7.08 – 7.05 (m, 1H, W^KR^-C^5^**H**),6.97 (t, ^3^*J* = 7.6 Hz, ^4^*J* = 7.4 Hz, 1H, W^KR^-C^6^**H**), 6.90 (s, 3H, R-N**H**_3_^+^), 4.91 – 4.84 (m, 2H, W^KR^-C^α^**H**, K-C^α^**H**), 4.81 (td, ^3^*J* = 7.8 Hz, ^3^*J* = 5.3 Hz, 1H, W^pA^-C^α^**H**), 4.56 (dq, ^3^*J* = 7.2 Hz,^3^*J* = 7.0 Hz, 1H, A-C^α^**H**), 4.45 (ddd, ^3^*J* = 7.8 Hz, ^3^*J* = 7.5 Hz, ^3^*J* = 7.5 Hz, 1H, R-C^α^**H**), 4.39 – 4.32 (m, 3H, L-C^α^**H**, D-C^α^**H**, p-C^α^**H**), 3.90 (dd, ^2^*J* = 15.4 Hz, ^3^*J* = 5.2 Hz, 1H, G-C^α^**H**_2_), 3.77 (dd, ^2^*J* = 15.4 Hz, ^3^*J* = 5.6 Hz, 1H, G-C^α^**H**_2_), 3.28 (dt, ^2^*J* = 10.0 Hz, ^3^*J* = 7.0 Hz, 1H, p-C^δ^**H**_2_),3.21 – 3.05 (m, 6H, R-C^δ^**H**_2_, W^KR^-C^β^**H**_2_, W^pA^-C^β^**H**_2_), 2.80 – 2.75 (m, 1H, p-C^δ^**H**_2_), 2.72 – 2.65 (m, 3H, K-C^ε^**H**_2_, D-C^β^**H**_2_), 2.57 (dd, ^2^*J* = 16.6 Hz, ^3^*J* = 9.5 Hz, 1H, D-C^β^**H**_2_), 2.52 – 2.51 (m, 1H, R-N^ε^**H**), 2.43 (s, 3H, K-NC**H**_3_), 1.84 – 1.76 (m, 3H, K-C^β^**H**2, p-C^β^**H**_2_), 1.75 – 1.69 (m, 1H, R-C^β^**H**_2_), 1.62 – 1.57 (m, 1H, p-C^β^**H**_2_), 1.57 – 1.48 (m, 4H, R-C^β^**H**_2_, L-C^β^**H**_2_, L-C^γ^**H**), 1.47 – 1.37 (m, 4H, K-C^δ^**H**_2_, R-C^γ^**H**_2_), 1.31 – 1.20 (m, 2H, p-C^γ^**H**_2_), 1.25 (d, ^3^*J* = 7.0 Hz, 1H, A-C**H**_3_), 1.11 – 0.99 (m, 2H, K-C^γ^**H**_2_), 0.84 (dd, ^3^*J* = 12.5 Hz, ^3^*J* = 6.0 Hz, 6H, L-C**H**_3_).

**^13^C-NMR** (DMSO-d6, 151 MHz) 𝛿 [ppm] = 173.4, 172.6, 172.1, 172.0, 171.9, 171.3, 170.9, 170.0, 169.8, 169.3, 157.1, 137.4, 136.6, 127.8, 126.8, 125.3, 124.8, 121.7, 121.3, 120.4, 118.5, 116.4, 114.5, 114.3, 111.7, 110.6, 110.1, 56.3, 56.1, 52.6, 52.4, 52.0, 50.7, 48.9, 47.2, 42.9, 41.3, 40.8, 39.1, 38.5, 35.9, 30.4, 29.8, 29.0, 28.6, 27.0, 26.5, 25.7, 25.1, 24.7, 23.8, 22.6, 22.2, 18.1.

| **ESI-MS** (*m/z*): | Calculated  [M^79Br^+H]^+^: 1202.5  [M^81Br^+H]^+^: 1204.5 | Determined [M^79Br^+H]^+^: 1202.5  [M^81Br^+H]^+^: 1204.5 |
| --- | --- | --- |
| **HR-MS** (*m/z*): | Calculated  [M^79Br^+H]^+^: 1202.5267  [M^81Br^+H]^+^: 1204.5261 | Determined [M^79Br^+H]^+^: 1202.5264  [M^81Br^+H]^+^: 1204.5246 |

#### c(DLAW^(6-Br)^p^(NMe)^KW^(6-Br)^RG) × 2 TFA (**11**)

**^1^H-NMR** (DMSO-d6, 273 K, 600 MHz) 𝛿 [ppm] = 12.50 (s, 1H, O**H**), 11.09 (s, 1H, W^pA^-N^1^**H**), 10.93 (s, 1H, W^KR^-N^1^**H**), 8.71 (s, 1H, R-N^α^**H**), 8.55 (d, ^3^*J* = 6.7 Hz, 1H, D-N^α^**H**), 8.07 (d, ^3^*J* = 7.1 Hz, 1H, A-N^α^**H**), 7.85 (s, 1H, G-N^α^**H**), 7.82 (d, ^3^*J* = 7.8 Hz, 1H, W^pA^-N^α^**H**), 7.73 (d, ^3^*J* = 8.4 Hz, 1H, W^pA^-C^4^**H**), 7.66 (d, ^3^*J* = 8.7 Hz, 1H, L-N^α^**H**), 7.63 (t, ^3^*J* = 5.8 Hz, ^3^*J* = 5.8 Hz, 3H, K-N**H**_3_^+^), 7.54 (d, ^4^*J* = 1.8 Hz, 1H, W^pA^-C^7^**H**), 7.53 (d, ^4^*J* = 1.7 Hz, 1H, W^KR^-C^7^**H**), 7.50 (d, ^3^*J* = 8.6 Hz, 1H, W^KR^-C^4^**H**), 7.52 – 7.49 (m, 1H, R-C=N**H**), 7.39 (d, ^3^*J* = 9.0 Hz, 1H, W^KR^-N^α^**H**), 7.25 (d, ^4^*J* = 2.3 Hz, 1H, W^pA^-C^2^**H**), 7.22 (d, ^4^*J* = 2.2 Hz, 1H, W^KR^-C^2^**H**), 7.09 (dd, ^3^*J* = 8.6 Hz, ^4^*J* = 1.7 Hz, 1H, W^KR^-C^5^**H**), 7.08 (dd, ^3^*J* = 8.4 Hz, ^4^*J* = 1.8 Hz, 1H, W^pA^-C^5^**H**), 6.90 (s, 3H, R-N**H**_3_^+^), 4.90 – 4.82 (m, 2H, W^KR^-C^α^**H**, p-C^α^**H**), 4.79 (td, ^3^*J* = 7.8 Hz, ^3^*J* = 5.2 Hz, 1H, W^pA^-C^α^**H**), 4.53 (dq, ^3^*J* = 7.1 Hz, ^3^*J* = 7.0 Hz, 1H, A-C^α^**H**), 4.44 (ddd, ^3^*J* = 7.6 Hz, ^3^*J* = 7.5 Hz, ^3^*J* = 7.5 Hz, 1H, R-C^α^**H**), 4.39 – 4.31 (m, 3H, L-C^α^**H**, D-C^α^**H**, K-C^α^**H**), 3.91 (dd, ^2^*J* = 15.5 Hz, ^3^*J* = 4.8 Hz, 1H, G-C^α^**H**_2_), 3.77 (dd, ^2^*J* = 15.5 Hz, ^3^*J* = 5.7 Hz, 1H, G-C^α^**H**_2_), 3.29 (ddd, ^2^*J* = 9.7 Hz, ^3^*J* = 7.0 Hz, ^3^*J* = 7.0 Hz, 1H, p-C^δ^**H**_2_), 3.17 – 3.03 (m, 6H, R-C^δ^**H**_2_, W^KR^-C^β^**H**_2_, W^pA^-C^β^**H**_2_), 2.86 – 2.77 (m, 1H, p-C^δ^**H**_2_), 2.72 – 2.63 (m, 3H, K-C^ε^**H**_2_, D-C^β^**H**_2_), 2.57 (dd, ^2^*J* = 16.7 Hz, ^3^*J* = 9.4 Hz, 1H, D-C^β^**H**_2_), 2.52 – 2.49 (m, 1H, R-N^ε^**H**), 2.41 (s, 3H, K-NC**H**_3_), 1.86 – 1.68 (m, 4H, K-C^β^**H**_2_, p-C^β^**H**_2_, R-C^β^**H**_2_), 1.67 – 1.46 (m, 6H, R-C^β^**H**_2_, p-C^β^**H**_2_, p-C^γ^**H**_2_, L-C^β^**H**_2_, L-C^γ^**H**), 1.48 – 1.37 (m, 4H, K-C^δ^**H**_2_, R-C^γ^**H**_2_), 1.31 – 1.25 (m, 1H, p-C^γ^**H**_2_), 1.24 (d, ^3^*J* = 7.0 Hz, 1H, A-C**H**_3_), 1.09 – 1.02 (m, 2H, K-C^γ^**H**_2_), 0.84 (dd, ^3^*J* = 12.5 Hz, ^3^*J* = 5.9 Hz, 6H, L-C**H**_3_).

**^13^C-NMR** (DMSO-d6, 151 MHz) 𝛿 [ppm] = 172.9, 172.0, 171.9, 171.6, 171.5, 170.4, 170.0, 169.5, 169.3, 168.8, 156.6, 137.0, 136.9, 126.4, 126.3, 125.6, 124.8, 121.2, 120.9, 120.8, 119.9, 114.0, 113.9, 113.8, 113.7, 110.5, 109.7, 55.8, 55.6, 52.2, 51.9, 51.5, 50.2, 48.4, 46.7, 42.4, 40.8, 40.3, 40.1, 38.6, 37.2, 35.4, 29.9, 29.4, 28.2, 26.5, 26.0, 25.2, 24.6, 24.2, 23.3, 22.1, 21.7, 17.6.

| **ESI-MS** (*m/z*): | Calculated  [M^79Br^+H]^+^: 1280.4  [M^81Br^+H]^+^: 1284.4 | Determined [M^79Br^+H]^+^: 1280.4  [M^81Br^+H]^+^: 1284.4 |
| --- | --- | --- |
| **HR-MS** (*m/z*): | Calculated  [M^79Br^+H]^+^: 1280.4372  [M^81Br^+H]^+^: 1284.4357 | Determined [M^79Br^+H]^+^: 1202.4378  [M^81Br^+H]^+^: 1284.4336 |

#### c(DLA^(6-Cl)^Wp(NMe)K^(6-Br)^WRG) × 2 TFA (**12**)

| **HR-MS** (*m/z*): | **Calculated** | **Measured** |
| --- | --- | --- |
|  | [M+H]^+^ 1236.47151 | [M+H]^+^ 1236.4797 |
|  | [M+Na]^+^ 1258.45346 | [M+Na]^+^ 1258.4536 |
|  |  |  |

#### c(DLA^(6-Cl)^Wp^(NMe)(Az)^ARG) × 2 TFA (**13**)

**^1^H-NMR**(600 MHz, DMSO-d_6_) δ[ppm] = 12.51 (s, 1H, O**H^Asp^**), 11.08 (d, ^3^*J* = 3.9 Hz, 1H, N**H^Indole^**), 8.76-8.70 (m, 1H, N**H**), 8.64 (d, ^3^*J*= 7.2 Hz, 1H, N**H**), 8.52 (d, ^3^*J* = 9.7 Hz, 1H, N**H**), 8.32 (d, ^3^*J* = 9.3 Hz, 1H, N**H**), 8.13 (s, 1H, N**H**), 7.89 (d, ^3^*J* = 7.6 Hz, 1H, N**H**), 7.80 (d, ^3^*J*=3.8 Hz, 1H, N**H**), 7.71 – 7.63 (m, 3H), 7.63–7.58 (m, 2H), 7.54 – 7.48 (m, 2H), 7.36 (d, ^3^*J*= 1.9 Hz, 1H), 7.33 (d, ^3^*J*= 3.8 Hz, 1H), 7.27 (d, ^3^*J* = 2.3 Hz, 1H), 7.15 (t, ^3^*J* =9.7 Hz, ^3^*J* = 9.7 Hz, 1H), 7.08 (t, ^3^*J* = 9.8 Hz, ^3^*J*=9.8 Hz, 1H), 6.93 (dd, ^3^*J* = 8.4 Hz, ^4^*J* = 1.9 Hz, 1H), 5.05 – 4.97 (m, 1H, C^α^**H**), 4.86 – 4.77 (m, 2H, C^α^**H**), 4.61-4.57 (m, 1H, C^α^**H**), 4.49 (q, ^3^*J* = 7.6 Hz, 1H, C^α^**H**), 4.41 – 4.30 (m, 2H, C^α^**H**), 4.27 (t, ^3^*J* = 6.9 Hz, ^3^*J* = 6.9 Hz, 1H, C^α^**H**), 3.96 – 3.89 (m, 1H, C^α^**H**_2_**^Gly^**), 3.79 (dd, ^2^*J*=15.2 Hz, ^3^*J* = 5.9 Hz, 1H, C^α^**H**_2_**^Gly^**), 3.56 – 3.48 (m, 1H, C^δ^**H**_2_**^pro^**), 3.31 – 3.25 (m, 1H), 3.20 – 3.04 (m, 4H), 2.84-2.80 (m, 1H, C**H**_2_**^Phe^**), 2.70 – 2.63 (m, 3H), 2.63 – 2.54 (m, 1H), 2.23 (s, 3H, NC**H_3_^Lys^**), 1.86 – 1.68 (m, 4H), 1.62- 1.47 (m, 5H), 1.47 – 1.35 (m, 4H), 1.28 (d, ^3^*J* = 6.9 Hz, 3H, C**H_3_^Ala^**), 1.07-0.97 (m, 2H), 0.84 (dd, ^2^*J*= 14.8 Hz, ^3^*J*= 5.7 Hz, 6H, C**H_3_^Leu^**).

**^13^C-NMR**(151 MHz, DMSO-d_6_) δ[ppm]= 172.9, 171.9, 171.7, 171.6, 170.4, 169.6, 169.2, 168.9, 156.7, 140.4, 138.3, 137.6, 136.4, 136.3, 136.1, 134.0, 126., 125.9, 125.8, 125.0, 122.3, 121.7, 119.5, 118.6, 116.5, 111.2, 109.5, 55.7, 53.3, 52.2, 51.6, 48.4, 46.8, 42.4, 38.6, 35.4, 29.9, 28.1, 26.5, 26.0, 25.2, 24.7, 24.2, 23.3, 22.1, 21.7, 21.5, 17.7.

| **ESI-MS** (*m/z*) | Calculated | Determined |
| --- | --- | --- |
|  | [M+H]^+^ 1169.565 | [M+H]^+^ 1169.60 |
|  | [M+2H]^2+^ 585.286 | [M+2H]^2+^ 585.33 |

#### c(DLA^(6-Br)^Wp^(NMe)^K^(Az)^ARG) × 2 TFA (**14**)

**^1^H-NMR**(600 MHz, DMSO-d_6_) δ[ppm]= 12.51 (s, 1H, O**H^Asp^**), 11.08 (t, ^3^*J*=2.9 Hz, ^3^*J*=2.9 Hz, 1H, N**H^Indole^**), 8.75- 8.71 (m, 1H, N**H**), 8.64 (t, ^3^*J* = 5.0 Hz, ^3^*J*= 5.0 Hz, 1H, N**H**), 8.51 (d, ^3^*J*= 9.6 Hz, 1H, N**H**), 8.32 (d, ^3^*J*= 9.4 Hz, 1H, N**H**), 8.13 (d, ^3^*J* = 6.5 Hz, 1H, N**H**), 7.89 (d, ^3^*J*= 7.5 Hz, 2H, N**H**), 7.79 (d, ^3^*J*= 3.8 Hz, 1H, N**H**), 7.72- 7.54 (m, 5H), 7.53 -7.45 (m, 3H), 7.35 - 7.29 (m, 1H), 7.26 (d, ^3^*J*= 2.4 Hz, 1H), 7.15 (t, ^3^*J* = 9.7 Hz, ^3^*J*= 9.7 Hz, 1H), 7.11 - 7.00 (m, 2H), 5.05 -4.97 (m, 1H, C^α^**H**), 4.86 - 4.77 (m, 2H, C^α^**H**), 4.62 - 4.56 (m, 1H, C^α^**H**), 4.49 (q, ^3^*J* = 7.6 Hz, ^3^*J*= 7.6 Hz, ^3^*J* = 7.6 Hz, 1H, C^α^**H**), 4.41 - 4.30 (m, 2H), 4.28 (t, ^3^*J* = 6.7 Hz, ^3^*J* = 6.7 Hz, 1H, C^α^**H**), 3.97- 3.89 (m, 1H, C^α^**H**_2_**^Gly^**), 3.79 (dd, ^2^*J*= 15.2 Hz, ^3^*J*=5.9 Hz, 1H, C^α^**H**_2_**^Gly^**), 3.55- 3.47 (m, 1H, C^δ^**H**_2_**^pro^**), 3.33 – 3.26 (m, 1H), 3.20 - 3.02 (m, 4H), 2.83 (s, 1H), 2.72- 2.64 (m, 3H), 2.58 (dd, ^2^*J* = 16.7 Hz, ^3^*J* = 9.5 Hz, 1H), 2.23 (s, 3H, NC**H_3_^Lys^**), 1.87- 1.80 (m, 2H), 1.79- 1.69 (m, 1H), 1.63-1.47 (m, 5H), 1.46-1.36 (m, 3H), 1.28 (d, ^3^*J* = 6.9 Hz, 3H, C**H_3_^Ala^**), 1.01 (dd, ^2^*J* = 9.4 Hz, ^3^*J*=7.5 Hz, 2H), 0.84 (dd, ^2^*J*= 14.8 Hz, ^3^*J* = 5.7 Hz, 6H, C**H_3_^Leu^**).

**^13^C-NMR**(126 MHz, DMSO-d_6_) δ[ppm]= 172.9, 171.9, 171.7, 171.6, 170.4, 169.6, 169.2, 168.9, 156.7, 140.36, 138.3, 137.6, 136.9, 136.3, 136.1, 126.4, 125.9, 124.9, 122.3, 121.7, 121.2, 119.9, 116.5, 114.11, 11.9, 109.6, 55.7, 53.3, 52.2, 51.6, 50.2, 48.5, 46.8, 42.4, 41.0, 40.3, 38.6, 35.4, 28.1, 26.48, 25.99, 25., 24.7, 24.2, 23.3, 22.1, 21.7, 17.7.

| **ESI-MS (***m/z*): | Calculated | Measured |
| --- | --- | --- |
|  | [M+H]^+^ 1213.515 | [M+H]^+^ 1215.54 |
|  | [M+2H]^2+^ 607.261 | [M+2H]^2+^ 608.29 |

# Late stage modification of Peptides

## Suzuki-Miyaura-coupling

K_3_PO_4_ (10 eq), peptide (1 eq.) and boronic acid (3 eq) were dissolved in degassed DMF/H_2_O (2:1, 900 μL) in a pressure-sealed brown glass vial under argon atmosphere. A suspension of sSPhos (12.5 mol %) and Na_2_PdCl_4_ (5 mol %) in a mixture of DMF/water (500 μL) was added. After heating to 90 °C for around 2 hours (conversion was monitored via LCMS) the suspension was filtered through a plug of Celite and washed with methanol (10 mL). After removal of the solvent under reduced pressure, the crude product was purified by preparative RP-HPLC (60 min, 5 % to 95% ACN in H_2_O).

## Heck-reaction

Cyclic peptide (1 eq), K_2_CO_3_ (1.5 eq), styrene (5.0 eq) and trisodium 3,3′,3′′-phosphanetriyltri(benzene-1-sulfonate) (TPPTS) (0.3 eq) were dissolved in degassed mixture of water and 1,4-dioxane (1:1, 2.5 mL) in a pressure-sealed brown glass under argon atmosphere. A freshly prepared solution of Pd(OAc)_2_ (0.1 eq) in 1,4-dioxane (100 μL) was added and the reaction was stirred for 2 hours at 100 °C under argon. Conversion was monitored by LC-MS. The resulting suspension was filtered through a plug of Celite and thoroughly washed with methanol (10 mL). After removal of the solvent under reduced pressure, the crude product was purified by preparative RP-HPLC (60 min, 5 to 95% ACN in H_2_O).

#### c(DLA^(6-(4-vinyl-benzoate)^Wp^(NMe)^KFRG) × 2 TFA (**15**)

| **ESI-MS** (*m/z*): | Calculated | Determined |
| --- | --- | --- |
|  | [M+H]^+^ 1231.625 | [M+H]^+^ 1231.61 |
|  | [M+2H]^2+^ 616.316 | [M+2H]^2+^ 616.31 |

#### c(DLA^(6-(9-anthracenyl)^Wp^(NMe)^KFRG) × 2 TFA (**16**)

| **ESI-MS** (*m/z*): | Calculated | Determined |
| --- | --- | --- |
|  | [M+H]^+^ 1261.651 | [M+H]^+^ 1261.64 |
|  | [M+2H]^2+^ 631.329 | [M+2H]^2+^ 631.33 |

#### c(DLAW^(5-(1-pyrenyl)^p^(NMe)^KFRG) × 2 TFA (**17**)

**^1^H-NMR** (DMSO-d6, 600 MHz) 𝛿 [ppm] = 12.49 (s, 1H, O**H**), 11.21 (d, ^4^*J* = 2.4 Hz, 1H, W-N^1^**H**), 8.62 – 8.55 (m, 2H, R-N^α^**H**, D-N^α^**H**), 8.37 (d, ^3^*J* = 7.7 Hz, 1H, W-C^5^-C**H**^pyr^), 8.34 (d, ^3^*J* = 7.7 Hz, 1H, W-C^5^-C**H**^pyr^), 8.29 – 8.21 (m, 3H, W-C^5^-C**H**^pyr^), 8.16 – 8.11 (m, 2H, W-C^5^-C**H**^pyr^), 8.10 (t, ^3^*J* = 7.7 Hz, 1H, W-C^5^-C**H**^pyr^), 8.08 (d, ^3^*J* = 7.7 Hz, 1H, W-C^5^-C**H**^pyr^), 7.88 – 7.83 (m, 2H, G-N^α^**H**, A-N^α^**H**), 7.71 (d, ^3^*J* = 7.3 Hz, 1H, W-N^α^**H**), 7.69 (s, 1H, W-C^4^**H**), 7.61 (d, ^3^*J* = 8.2 Hz, 1H, W-C^7^**H**), 7.59 – 7.58 (m, 1H, R-C=N**H**), 7.57 – 7.53 (m, 3H, L-N^α^**H**, K-N**H**_3_^+^), 7.45 (d, ^3^*J* = 2.4 Hz, 1H, W-C^2^**H**), 7.44 – 7.40 (m, 1H, F-N^α^**H**), 7.39 (dd, ^3^*J* = 8.2 Hz, ^4^*J* = 1.6 Hz, 1H, W-C^6^**H**), 7.21 – 7.14 (m, 3H, F-C**H*^meta^,*** F-C**H*^para^***), 7.08 (d, ^3^*J* = 7.7 Hz, 2H, F-C**H*^ortho^***), 6.74 (s, 3H, R-N**H**_3_^+^), 4.77 (dd, ^3^*J* = 7.3 Hz, ^3^*J* = 6.7 Hz, 1H, W-C^α^**H**), 4.72 – 4.64 (m, 2H, F-C^α^**H**, K-C^α^**H**), 4.44 (dq, ^3^*J* = 7.0 Hz, ^3^*J* = 6.9 Hz, 1H, A-C^α^**H**), 4.40 (dd, ^3^*J* = 7.7 Hz, *J* = 7.6 Hz, 1H, R-C^α^**H**), 4.33 – 4.35 (m, 2H, L-C^α^**H**, D-C^α^**H**), 4.09 – 4.03 (m, 1H, p-C^α^**H**), 4.00 (dd, ^2^*J* = 15.4 Hz, ^3^*J* = 5.4 Hz, 1H, G-C^α^**H**_2_), 3.68 (dd, ^2^*J* = 15.4 Hz, ^3^*J* = 4.9 Hz, 1H, G-C^α^**H**_2_), 3.29 – 3.22 (m, 2H, W-C^β^**H**2, p-C^δ^**H**_2_), 3.14 (dd, ^3^*J* = 14.1 Hz, ^3^*J* = 9.3 Hz, 1H, W-C^β^**H**_2_), 3.05 (dt, ^3^*J* = 7.2 Hz, ^3^*J* = 7.1 Hz 2H, R-C^δ^**H**_2_), 2.97 – 2.92 (m, 2H, F-C^β^**H**_2_), 2.78 – 2.70 (m, 3H, F-C^β^**H**2, K-C^ε^**H**_2_), 2.67 – 2.54 (m, 3H, p-C^δ^**H**2, D-C^β^**H**_2_), 2.53 – 2.51 (m, 1H, R-N^ε^**H**), 2.50 (s, 3H, K-NC**H**_3_), 1.77 – 1.71 (m, 2H, R-C^β^**H**_2_, p-C^γ^**H**_2_), 1.70 - 1.63 (m, 2H, R-C^β^**H**_2_, p-C^γ^**H**_2_), 1.62 – 1.54 (m, 3H, p-C^β^**H**_2_, K-C^β^**H**_2_), 1.53 – 1.41 (m, 4H, L-C^β^**H**_2_, L-C^γ^**H**, p-C^β^**H**_2_), 1.40 – 1.33 (m, 2H, R-C^γ^**H**_2_), 1.32 - 1.22 (m, 2H, K-C^δ^**H**_2_), 1.18 (d, ^3^*J* = 6.9 Hz, 1H, A-C**H**_3_), 1.03 – 0.94 (m, 2H, K-C^γ^**H**_2_), 0.81 (dd, ^3^*J* = 9.9 Hz, ^3^*J* = 5.9 Hz, 6H, L-C**H**_3_).

| **ESI-MS** (*m/z*): | Calculated  [M+H]^+^: 1285.7 | Determined  [M+H]^+^: 1285.7 |
| --- | --- | --- |
| **HR-MS** (*m/z*): | Calculated  [M+H]^+^: 1285.6522 | Determined  [M+H]^+^: 1285.6485 |

#### c(DLAW^(5-(3-aminophenyl))^p^(NMe)^KFRG) × 3 TFA (**18**)

**^1^H-NMR** (DMSO-d6, 600 MHz) 𝛿 [ppm] = 11.01 (s, 1H, W-N^1^**H**), 8.65 – 8.61 (m, 2H, R-N^α^**H**, D-N^α^**H**), 7.92 (d, ^3^*J* = 6.9 Hz, 1H, A-N^α^**H**), 7.88 (dd, ^3^*J* = 5.1 Hz, ^3^*J* = 5.1 Hz, 1H, G-N^α^**H**), 7.73 (d, ^3^*J* = 7.4 Hz, 1H, W-N^α^**H**), 7.70 (d, ^4^*J* = 1.6 Hz, 1H, W-C^4^**H**), 7.65 (d, ^3^*J* = 9.2 Hz, 1H, L-N^α^**H**), 7.64 – 7.60 (m, 3H, K-N**H**_3_^+^), 7.54 (dd, 1H, ^4^*J* = 5.9 Hz, ^4^*J* = 5.9 Hz, R-C=N**H**), 7.43 (d, ^3^*J* = 8.4 Hz, 1H, W-C^7^**H**), 7.37 (d, ^3^*J* = 9.3 Hz, 1H, F-N^α^**H**), 7.32 (dd, ^3^*J* = 8.4 Hz, ^4^*J* = 2.0 Hz, 1H, W-C^6^**H**), 7.30 (d, ^4^*J* = 2.0 Hz, 1H, W-C^2^**H**), 7.27 – 7.17 (m, 7H, F-C**H*^ortho^***, F-C**H*^meta^,*** F-C**H*^para^***, W-C^5^-Ph-**H*^ortho^***), 7.13 – 7-08 (m, 1H, W-C^5^-Ph-**H*^meta^***), 6.86 (s, 3H, R-N**H**_3_^+^), 6.78 (s, 1H, W-C^5^-Ph-**H*^para^***), 4.83 – 4.76 (m, 3H, F-C^α^**H**, p-C^α^**H**, W-C^α^**H**), 4.47 (dq, ^3^*J* = 7.1 Hz, ^3^*J* = 6.9 Hz, 1H, A-C^α^**H**), 4.42 (ddd, ^3^*J* = 7.7 Hz, ^3^*J* = 7.6 Hz, ^3^*J* = 7.6 Hz, 1H, R-C^α^**H**), 4.37 – 4.28 (m, 2H, L-C^α^**H**, D-C^α^**H**), 4.23 (dd, ^3^*J* = 7.0 Hz, ^3^*J* = 7.0 Hz, 1H, K-C^α^**H**), 4.01 (dd, ^2^*J* = 15.5 Hz, ^3^*J* = 5.1 Hz, 1H, G-C^α^**H**_2_), 3.71 (dd, ^2^*J* = 15.5 Hz, ^3^*J* = 5.1 Hz, 1H, G-C^α^**H**_2_), 3.36 – 3.29 (m, 1H, p-C^δ^**H**_2_), 3.24 – 3.17 (m, 1H, W-C^β^**H**_2_), 3.16 – 3.03 (m, 4H, W-C^β^**H**_2_, F-C^β^**H**_2_, R-C^δ^**H**_2_), 2.91 – 2.83 (m, 2H, F-C^β^**H**_2_, p-C^δ^**H**_2_), 2.71 – 2.63 (m, 3H, K-C^ε^**H**_2_, D-C^β^**H**_2_), 2.57 (dd, ^2^*J* = 16.7 Hz, ^3^*J* = 9.6 Hz, 1H, D-C^β^**H**_2_), 2.52 – 2.49 (m, 1H, R-N^ε^**H**), 2.20 (s, 3H, K-NC**H**_3_), 1.82 – 1.62 (m, 4H, K-C^β^**H**_2_, p-C^β^**H**_2_, R-C^β^**H**_2_), 1.58 – 1.46 (m, 5H, L-C^β^**H**_2_, L-C^γ^**H**, K-C^β^**H**_2_, R-C^β^**H**_2_), 1.45 – 1.34 (m, 4H, K-C^δ^**H**_2_, R-C^γ^**H**_2_), 1.25 (d, ^3^*J* = 7.1 Hz, 1H, A-C**H**_3_), 1.22 – 1.16 (m, 2H, p-C^γ^**H**_2_), 1.05 – 0.96 (m, 2H, K-C^γ^**H**_2_), 0.84 (dd, ^3^*J* = 10.5 Hz, ^3^*J* = 5.8 Hz, 6H, L-C**H**_3_).

**^13^C-NMR** (DMSO-d6, 151 MHz) 𝛿 [ppm] = 172.9, 171.5, 171.4, 171.3, 170.7, 170.4, 169.7, 169.4, 168.8, 156.6, 143.1, 137.8, 135.7, 129.6, 129.5, 129.4, 129.2, 128.0, 127.8, 126.2, 124.9, 120.4, 119.9, 116.2, 115.7, 111.9, 109.5, 103.9, 55.7, 55.5, 53.3, 52.0, 51.7, 50.2, 48.5, 46.7, 42.2, 40.7, 40.3, 40.0, 38.6, 38.2, 35.4, 29.8, 29.6, 28.0, 26.5, 26.0, 25.2, 24.6, 24.2, 23.3, 22.2, 21.7, 17.8.

| **ESI-MS** (*m/z*): | Calculated  [M+H]^+^: 1176.6 | Determined  [M+H]^+^: 1176.6 |
| --- | --- | --- |
| **HR-MS** (*m/z*): | Calculated  [M+H]^+^: 1176.6318 | Determined  [M+H]^+^: 1176.6318 |

#### c(DLA^(5-Cl)^Wp(NMe)K^(6-Br)^WRG) × 2 TFA (**19**)

**^1^H-NMR** (600 MHz, DMSO-d_6_) δ[ppm]= 12.39 (s, 1H, O**H^Asp^**), 11.09 (d, ^3^*J* = 2.6 Hz, 1H, N**H^Indole^**), 10.80(d, ^3^*J*= 2.3 Hz, 1H, N**H^Indole^**); 8.63 (d. ^3^*J*= 8.0 Hz, 1H), 8.51 (d, ^3^*J* = 6.6 Hz, 1H), 7.97 (d, ^3^*J* = 5.5 Hz, 1H, N**H^Gly^**), 7.91 (d, ^3^*J* = 6.9 Hz, 1H, N**H^Ala^**), 7.66 (d, ^3^*J*= 8.4 Hz, 1H), 7.62- 7.57 (m, 3H), 7.55- 7.49 (m, 1H), 7.49- 7.43 (m, 2H), 7.36 (d, ^3^*J* = 8.9 Hz, 1H), 7.29 (d, ^3^*J* = 8.6 Hz, 1H), 7.25 (d, ^3^*J*= 2.5 Hz, 1H, N**H^Indole-2^**), 7.02- 6.98 (m, 2H), 4.80- 4.74 (m, 2H), 4.68- 4.61 (m, 1H), 4.45 – 4.30 (m, 2H, C**^α^H^Ala^**), 4.30- 4.22 (m, 2H), 4.08 (dd, ^3^*J*= 7.7 Hz, ^3^*J*= 5.8 Hz, 1H), 3.85 (dd, ^2^*J* = 15.5 Hz, ^3^*J*= 4.8 Hz, 1H, C**^α^H**_2_**^Gly^**), 3.66 (dd, ^2^*J*= 15.5 Hz, ^3^*J*= 5.7 Hz, 1H, C**^α^H**_2_**^Gly^**), 3.23 – 3.10 (m, 1H), 3.08 – 2.91 (m, 4H), 2.67 – 2.53 (m, 4H), 2.49 (dd, ^2^*J*= 16.7 Hz, ^3^*J* = 9.6 Hz, 1H), 2.38 (s, 3H, NC**H_3_^Lys^**), 1.75 – 1.60 (m, 3H), 1.58- 1.25 (m, 8H, C**H^Leu^**(CH_3_)_2_), 1.17 (d, ^3^*J*= 6.9 Hz, 3H, C**H_3_^Ala^**), 1.13 - 1.03 (m, 1H), 1.01- 0.88 (m, 1H), 0.75 (dd, ^2^*J*= 10.9 Hz, ^3^*J* = 5.8 Hz, 6H, C**H_3_^Leu^**).

**^13^C-NMR** (151 MHz, DMSO-d_6_) δ[ppm]= 173.3, 172.5, 172.2, 172.0, 172.0, 171.9, 171.4, 171.0, 170.1, 169.9, 169.2, 168.8, 167.6, 159.2, 159.0, 158.8, 158.6, 157.3, 157.1, 137.7, 137.6, 135.0, 128.9, 126.8, 126.7, 126.6, 126.3, 125.9, 123.6, 121.8, 121.5, 121.4, 121.2, 120.7, 117.8, 114.5, 114.4, 114.3, 114.2, 113.6, 110.9, 109.6, 107.6, 56.3, 56.1, 52.9, 52.6, 52.5, 52.4, 52.2, 50.7, 49.0, 47.2, 42.8, 35.9, 30.6, 28.6, 27.7, 27.0, 26.5, 25.7, 25.3, 25.1, 24.7, 23.8, 22.6, 22.1, 18.1.

| **HR-MS** (*m/z*): | Calculated | Measured |
| --- | --- | --- |
|  | [M+H]^+^ 1236.47151 | [M+H]^+^ 1236.4709 |
|  | [M+2H]^2+^ 618.73939 | [M+2H]^2+^ 618.7401 |

#### c(DLAFp^(NMe)^K^(6-(9-anthracenyl)^WRG) × 2 TFA (**20**)

| **ESI- MS** (*m/z*): | Calculated | Measured |
| --- | --- | --- |
|  | [M+H]^+^ 1261.652 | [M+H]^+^ 1261.69 |
|  | [M+2H]^2+^ 631.329 | [M+2H]^2+^ 631.36 |
| **HR- MS** (*m/z*): | Calculated | Measured |
|  | [M+H]^+^ 1261.65167 | [M+H]^+^ 1261.6490 |
|  | [M+2H]^2+^ 631.32948 | [M+2H]^2+^ 631.3297 |

#### c(DLA^(5-Cl)^Wp(NMe)K^(6-(9-anthracenyl)^WRG) × 2 TFA (**21**)

| **ESI-MS** (*m/z*): | Calculated | Measured |
| --- | --- | --- |
|  | [M+H]^+^ 1334.623 | [M+H]^+^ 1334.64 |
|  | [M+2H]^2+^ 667.815 | [M+2H]^2+^ 677.83 |

#### c(DLA^(6-Cl)^Wp^(NMe)^K^(6-(9-anthracenyl)^WRG) × 2 TFA (**22**)

| **ESI-MS** (*m/z*): | Calculated | Measured |
| --- | --- | --- |
|  | [M+H]^+^ 1334.624 | [M+H]^+^ 1334.66 |
|  | [M+2H]^2+^ 667.815 | [M+2H]^2+^ 667.83 |
| **HR-MS** (*m/z*): | Calculated  [M+H]^+^ 1334.62360  [M+2H]^2+^ 667.81544 | Determined  [M+H]^+^ 1334.6223  [M+2H]^2+^ 667.8163 |

# Cell Culture and Bioassays

### Solid-phase binding assay (ELISA)

In a competitive ELISA using isolated integrins, the activity of each peptide was evaluated. Cilengitide (α_V_β_3_: 0.54 nM; α_5_β_1_: 15.4 nM), c(DLAFp(NMe)KFRG) (α_V_β_6_: 0.26 nM; α_V_β_8_: 23.6 nM), and tirofiban (α_IIb_β_3_: 1.2 nM) were used as reference standards. The assay was performed on flat-bottom 96-well immuno plates (Brand). Initially, the wells were coated at 4 °C with 100 µL of the respective native ligand (vitronectin for α_V_β_3_, fibronectin for α_5_β_1_, LAP–TGFβ for α_V_β_6_ and α_V_β_8_, and fibrinogen for α_IIb_β_3_, Table S2, Table S4) dissolved in carbonate buffer (15 mM Na_2_CO_3_, 35 mM NaHCO_3_, pH 9.6).

Table S2: Used coating material (native ligands) and their concentration for several Integrins.

| Integrin | Native Ligand |
| --- | --- |
| α_V_β_3_ | 1.0 μg/mL human vitronectin, R&D |
| α_5_β_1_ | 0.5 μg/mL human fibronectin, R&D |
| α_V_β_6_ | 0.4 μg/mL LAP (TGF-β), R&D |
| α_V_β_8_ | 0.4 μg/mL LAP (TGF-β), R&D |
| α_IIb_β_3_ | 10.0 μg/mL human fibrinogen, Merck Millipore |

Subsequently, the wells were washed with PBS-T buffer (3 × 200 µL/well) and blocked for 1 h at room temperature (RT) with TS-B buffer (150 µL/well).

Serial dilutions of the reference standards as well as the test compounds were prepared in TS-B buffer (1:5 or 1:10). After another washing step with PBS-T buffer (3 × 200 µL/well), the following solutions were added: row A was filled with 100 µL TS-B buffer as a negative control, rows B–G received 50 µL of the respective dilution series, and row H was filled with 50 µL TS-B buffer per well . Subsequently, the corresponding human integrin (Table S3), dissolved in TS-B buffer, was added to rows B–H (50 µL/well). The plate was then incubated for exactly 1 h at room temperature (RT).

Table S3: Proteins applied in the competitive ELISA.

| Integrin/Antibody | |
| --- | --- |
| α_V_β_3_ | 2.0 μg/mL, human α_v_β_3_ integrin, R&D |
| 1^st^ AB | 2.0 μg/mL, mouse anti-human CD51/61, BD Bioscience |
| 2^nd^ AB | 1.0 μg/mL, anti-mouse IgG-POD goat, Sigma-Aldrich |
|  | |
| α_5_β_1_ | 2.0 μg/mL, human α_5_β_1_ integrin, R&D |
| 1^st^ AB | 1.0 μg/mL, mouse anti-human CD49e, BD Bioscience |
| 2^nd^ AB | 2.0 μg/mL, anti-mouse IgG-POD goat, Sigma-Aldrich |
|  | |
| α_V_β_6_ and α_V_β_8_ | 0.5 μg/mL, human α_v_β_6_ or α_v_β_8_ integrin, R&D |
| 1^st^ AB | 1:1000 dilution in TS–B buffer, anti-α_v_ mouse anti-human CD51, Sigma-Aldrich |
| 2^nd^ AB | 2.0 μg/mL, anti-mouse IgG-POD goat, Sigma-Aldrich |
|  | |
| α_IIb_β_3_ | 5.0 μg/mL, human α_IIb_β_3_ integrin, Merck Millipore |
| 1^st^ AB | 2.0 μg/mL, mouse anti-human CD41b, BD Bioscience |
| 2^nd^ AB | 2.0 μg/mL, anti-mouse IgG-POD goat, Sigma-Aldrich |

Table S4: Used Buffer and reagents for competitive ELISA.

| TS-B-Buffer | 20 mM Tris-HCI 3.152 g, 1 L MPW, pH= 7.5  150 mM NaCl, 8.766 g; 1 mM CaCl∙2H_2_O, 147.06 mg;  1 mM MnCl_2_∙4H_2_O, 197.91 mg; 1 mM MgCl_2_∙6H_2_O, 203.30 mg.  Before every use BSA (1%) was added freshly. |
| --- | --- |
| Carbonate-Buffer | 1 L MPW, 15 mM Na_2_CO_3_ 1.59 g, 35 mM NaHCO_3_ 2.94 g  pH = 9.6 |
| PBS-T-Puffer | 1 L MPW, 140 mM NaCI 8.18 g, 2.7 mM KCl 200 mg,  10 mM Na_2_HPO_4_∙2H_2_O: 1.77 g  2 mM KH_2_PO_4_**,** 240 mg, pH = 7.4, 0.01 % Tween20 (100 μL) |

Subsequently, the wells were washed with PBS-T buffer (3 × 200 µL/well), and the primary antibody (100 µL/well, Table S3) was added and incubated for 1 h at RT. The wells were then washed again with PBS-T buffer (3 × 200 µL/well), followed by the addition of the secondary antibody (100 µL/well, Table S3) and incubation for another 1 h at RT. After a final washing step with PBS-T buffer, color development was performed using SeramunBlau® fast2 (Seramun Diagnostics GmbH, 50 µL/well). The reaction was stopped with 2 M aqueous H₂SO₄ (50 µL/well) once a blue color gradient became visible in the rows containing the internal standard (Cilengitide): α_V_β_3_:40 s; α_5_β_1_: 1.5 min; α_V_β_6_: 1.5 min; α_V_β_8_: 0.5 min; α_IIb_β_3_: 0.5 min.

The absorbance was measured with a TECAN plate reader at 450 nm and corrected by subtraction of the absorbance at 620 nm. IC_50_ values (inhibition concentration of 50% cell adhesion) for the tested compounds were determined with OriginPro 2016 (sigmoidal fit: DoseResp), with the inflection point of the fitted curve representing the IC₅₀-value. The compounds were tested once or multiple times in duplicate or triplicate measurements per plate.

### Cell culture und used cell lines

The human melanoma cell line HN (DSMZ German Collection, DSMZ no.: ACC417) overexpressing α_V_β_6_ is from an oral squamous cell carcinoma (origen: established from the tumor of a 60-year-old man with moderately differentiated squamous cell carcinoma of the soft palate which was invasive into muscle layer; cell line was established from a cervical lymph node metastasis which occurred seven years after initial treatment of the primary tumor; the tumor metastasized not only to the lungs, but also to the brain; described as anchorage-independent and tumorigenic in nude mice). Cells were cultured in 90% DMEM (4.5 g/L glucose) + 10-20% h.i. FBS, 20% h.i. FBS in the first week. (Storage: frozen with 70% medium, 20% FBS, 10% DMSO).

The epithelial melanoma cell line WM-115 (ATCC) overexpressing α_V_β_3_ was cultivated in MEM supplemented with 10 % FBS and gentamycin (50 μg/mL).

Cells were cultured at 37 °C under a humidified atmosphere with 5.3% CO_2_. To exclude any potential selection effects, all cell lines were maintained in antibiotic-free culture conditions.

### Flow cytometry for determination of integrin-expression levels

Cells were cultivated in a fibronectin coated cell culture flasks and incubated at 37 °C until the formation of a monolayer. The cells were detached with accutase (Pan Biotech), washed with medium and resuspended in PBS buffer (10^5^ cells/mL, 137 mM NaCl, 2.7 mM KCl, 10 mM Na_2_HPO_4_,2 mM KH_2_PO_4_, 300 μL).

Table S5: Used proteins for FACS measurement to determine the Integrin expression level.

| Integrin | Antibody | Protein |
| --- | --- | --- |
| α_V_ | 1st | 0.60 µL, MAB1978 mouse anti-human CD51, 0.5 mg/mL, Sigma Aldrich |
|  | 2nd | 1.25 µL, Alexa Fluor® 488 goat anti-mouse IgG (H+L), 2 mg/mL, Invitrogen |
| α_V_β_3_ | 1st | 0.60 µL, Mouse anti-human CD51/CD61, 0.5 mg/mL, BD Bioscience |
|  | 2nd | 1.25 µL, Alexa Fluor® 488 goat anti-mouse IgG (H+L), 2 mg/mL, Invitrogen |
| α_V_β_6_ | 1st | 1.60 µL, PE mouse anti-human integrin α_V_β_6_, 0.2 mg/mL, BD Bioscience |
|  | 2nd | 1.25 µL, Alexa Fluor® 488 goat anti-mouse IgG (H+L), 2 mg/mL, Invitrogen |
| α_V_β_8_ | 1st | 0.60 µL, Anti-integrin α_V_β_8_ rabbit anti-human, 0.3 mg/mL, ZooMAb® |
|  | 2nd | 1.25 µL, Alexa Fluor® 568 goat anti-rabbit IgG (H+L), 2 mg/mL, Invitrogen |
| α_5_β_1_ | 1st | 0.60 µL, Mouse anti-human CD49e, 0.5 mg/mL, BD Bioscience |
|  | 2nd | 1.25 µL, Alexa Fluor® 488 goat anti-mouse IgG (H+L), 2 mg/mL |

After incubation (15 min) of the first antibody at 4°C, cells were centrifuged (10 min, 1800 rpm/350 x g) and washed with PBS buffer (800 μL). After resuspending in 300 μL PBS-Puffer, the secondary antibody (Table S5) was added and incubation on ice was performed (15 min). After centrifugation and washing with PBS buffer, the cells were resuspended in PBS buffer (300 µl) and measured with a S3e Cell Sorter (BioRad) by excitation at 488 nm and 568 nm or a Beckman Coulter Gallio by excitation at 488 nm. For each sample, 30,000 events were measured. Pure cells and cells treated with secondary antibody were measured as controls.

### Flow cytometry for investigating cellular uptake

Cells were cultivated in a fibronectin coated cell culture flask and incubated at 37 °C until the formation of a monolayer. The cells were detached with accutase (Pan Biotech), washed with medium and resuspended (~10^5^ cells/mL) in PBS buffer (137 mM NaCl, 2.7 mM KCl, 10 mM Na_2_HPO_4_,2 mM KH_2_PO_4_, 300 μL) containing 10 μMol of the measured peptide. A solution of CaCl_2_, Mn Cl_2_ and Mg Cl_2_ (each 100 mM) in MPW was added with the same volume as PBS was added before incubating one hour at 37°C and at 4°C to test for non-selective cell uptake through the membrane. Untreated cells were used as reference. After centrifugation (10 min, 1800 rpm/350 x g) and washing with PBS buffer, the cells were resuspended in PBS buffer and measured with a a Beckman Coulter Gallio by excitation at 488 nm. For each sample, 30,000 events were measured.

### Crystal violet-based adhesion assay

96-well plates were coated with the natural ligand* by overnight incubation at 4 °C. Plates were washed with Phosphate Buffered Saline (PBS) solution and then blocked at 4°C for 1 h with PBS containing 5% v/v non-fat dry milk.

Cells were cultivated in a fibronectin coated cell culture flasks and incubated at 37 °C until the formation of a monolayer. The cells were detached with accutase (Pan Biotech), washed with medium and resuspended (~0.5 × 10^6^ cells/mL) in PBS buffer (137 mM NaCl, 2.7 mM KCl, 10 mM Na2HPO4, 2 mM KH2PO4, 300 μL).

HN cell suspensions were exposed to different amounts of compounds 1–3 (final concentration ranged from 10 μM to 50 nM). A preincubation at 37 °C for 30 min was performed to allow the ligand–receptor equilibrium to be reached. Assays were performed in the presence of 2 mM of MnCl_2_, MgCl_2_ and CaCl_2_. Cells were then plated on the coated plate (~5∙10^4^ cells/well) and incubated at 37 °C for 1 h. After incubation, plates were washed with PBS to remove the non-adherent cells, and 200 μL of 0.5 % crystal violet solution** was added. After 2 h of incubation at 4 °C, cells were washed with water (4x∙190 μL) permeabilized with 50 μL DMSO (20 min, shaking in the dark).

The absorbance was measured with a TECAN plate reader at 550 nm and corrected by subtraction of the absorbance at 620 nm. Experiments were conducted in triplicate and repeated at least twice.

IC_50_ values (inhibition concentration of 50% cell adhesion) for the tested compounds were determined with OriginPro 2016 (sigmoidal fit: DoseResp), with the inflection point of the fitted curve representing the IC₅₀-value.

*

- Fibronectin (0,1 μg/mL stock solution: 50 μL per 5mL for 4 rows (48 wells)
- LAP-TGF ß1 (0,2 μg/mL stock solution: 25 μL per 5mL for 4 rows (48 wells)

** Crystal violet first dissolved in water, then methanol was added. Solution was stored in dark for maximum two months at 7°C

# References

[1] A.-C. Moritzer, H. Minges, T. Prior, M. Frese, N. Sewald, H. H. Niemann, *J. Biol. Chem.* **2019**, *294*, 2529.
[2] M. Frese, P. H. Guzowska, H. Voß, N. Sewald, *ChemCatChem* **2014**, *6*, 1270.
[3] E. J. Watkins, P. J. Almhjell, F. H. Arnold, *Chembiochem* **2020**, *21*, 80.
[4] I. Kemker, C. Schnepel, D. C. Schröder, A. Marion, N. Sewald*, J. Med. Chem.* **2019**, *62*, 7417.
[5] Y. S. Moroz, W. Binder, P. Nygren, G. A. Caputo, I. V. Korendovych, *ChemComm* **2013**, *49*, 490.
[6] J. Chatterjee, B. Laufer, H. Kessler, *Nat. Protoc.* **2012**, *7*, 432.

# Appendix

#### Spectra

Figure S1: Absorption spectrum of **20** in water.

Figure S2: Fluorescence excitation and emission spectrum of **20** in water.

#### Flow cytometry for investigating cellular uptake of **19**

Figure S3: **A, B**: Flow cytometry analysis of HN-cells treated with **19**; **C**: FACS measurement:green: untreated HN-cells; red: HN-cells treated with **19** at 4°C, 1h; **blue**: HN-cells treated with **19** at 37°C, 1h

## NMR- and UV-Spectra

#### 6-Bromo-L-tryptophan

Figure S4: ^1^H-NMR-spectrum of 6-Bromo-L-tryptophan **1** in DMSO-d_6_

Figure S5: ^13^C-NMR-spectrum of 6-Bromo-L-tryptophan **1** in DMSO-d_6_

#### 6-Chloro-L-tryptophan

Figure S6: ^1^H-NMR-spectrum of 6-Chloro-L-tryptophan **2** in DMSO-d_6_

#### 7-Bromo-L-tryptophan

Figure S7: ^1^H-NMR-spectrum of 7-Bromo-L-tryptophan **3** in DMSO-d_6_

Figure S8: ^13^C-NMR-spectrum of 7-Bromo-L-tryptophan in DMSO-d_6._

Figure S9: UV traces at 220nm and 280 nm of 7-bromo-L-tryptophan**.**

#### 5-Bromo-L-tryptophan

Figure S10: UV traces at 220nm and 280 nm of 5-Bromo-L-tryptophan.

#### ß-(1-Azulenyl)-L-alanine

Figure S11: ^1^H-NMR-spectrum of ß-(1-Azulenyl)-L-alanine  in DMSO-d_6_

Figure S12: ^13^C-NMR-spectrum of ß-(1-Azulenyl)-L-alanine **6** in DMSO-d_6_

Figure S13: UV traces at 220nm and 280 nm of ß-(1-Azulenyl)-L-alanine.

#### Fmoc-5-Chloro-L-tryptophan

Figure S14: UV traces at 220nm and 280 nm of 5-Chloro-l-tryptophan.

#### Fmoc-ß-(1-Azulenyl)-L-alanine

Figure S15: UV traces at 220nm and 280 nm of Fmoc-ß-(1-Azulenyl)-L-alanine.

Figure S16: ^1^H-NMR-spectrum of Fmoc-ß-(1-Azulenyl)-L-alanine in DMSO-d_6_

Figure S17: ^13^C-NMR-spectrum of Fmoc-ß-(1-Azulenyl)-L-alanine in DMSO-d_6_

##### Fmoc-5-L-Bromtryptophan

Figure S18: 1H-NMR spectrum of Fmoc-5-L-bromotryptophan in DMSO-d6

Figure S19: RP-HPLC chromatogram (220 nm) of Fmoc-5-L-bromtryptophan.

#### Fmoc-6-Bromo-L-tryptophan

Figure S20: ^1^H-NMR-spectrum of Fmoc-6-bromo-l-tryptophan in DMSO-d_6_

#### Fmoc-6-Chloro-L-tryptophan

Figure S21: UV traces at 220nm and 280 nm of Fmoc-5-chloro-l-tryptophan.

#### Fmoc-7-Bromo-L-tryptophan

Figure S22: ^1^H-NMR-spectrum of Fmoc-7-bromo-l-tryptophan in DMSO-d_6_

#### Nonapeptides

#### c(DLAFp^(NMe)^K^(5-Br)^WRG) × 2 TFA (**1**)

Figure S23: ^1^H-NMR spectrum of **1** in DMSO-d6.

Figure S24: RP-HPLC chromatogram (220 nm) of **1**.

#### c(DLAFp^(NMe)^K^(6-Br)^WRG) × 2 TFA (**2**)

Figure S25: ^1^H-NMR spectrum of **2** in DMSO-d6.

Figure S26: RP-HPLC chromatogram (220 nm) of **2**.

#### c(DLAFp^(NMe)^K^(7-Br)^WRG) × 2 TFA (**3**)

Figure S27: ^1^H-NMR spectrum of **3** in DMSO-d6.

Figure S28: RP-HPLC chromatogram (220 nm) of **3**.

#### c(DLAWp^(NMe)^K^(6-Br)^WRG) × 2 TFA (**4**)

Figure S29: ^1^H-NMR spectrum of **4** in DMSO-d6.

Figure S30: RP-HPLC chromatogram (220 nm) of **4**.

c(DLA^(5-Cl)^Wp^(NMe)^KFRG) × 2 TFA (**5**)

Figure S31: UV traces at 220nm and 280 nm of c(DLAFp^(NMe)^K^(6-Br)^WRG) **5.**

#### c(DLA^(6-Cl)^Wp^(NMe)^KFRG) × 2 TFA (**6**)

Figure S32: ^1^H-NMR-spectrum of **6** in DMSO-d_6_

Figure S33: ^13^C-NMR-spectrum of **6** in DMSO-d_6_

Figure S34: UV traces at 220nm and 280 nm of **6**.

#### c(DLA^(5-Br)^Wp^(NMe)^KFRG) × 2 TFA (**7**)

Figure S35: ^1^H-NMR spectrum of **7** in DMSO-d6.

Figure S36: RP-HPLC chromatogram (220 nm) of **7**.

#### c(DLA^(6-Br)^Wp^(NMe)^KFRG) × 2 TFA (**8**)

Figure S37: ^1^H-NMR spectrum of **8** in DMSO-d6.

Figure S38: RP-HPLC chromatogram (220 nm) of **8**.

#### c(DLA^(7-Br)^Wp^(NMe)^KFRG) × 2 TFA (**9**)

Figure S39: ^1^H-NMR spectrum of **9** in DMSO-d6.

Figure S40: RP-HPLC chromatogram (220 nm) of **9**.

c(DLA^(6-Br)^Wp^(NMe)^KWRG) × 2 TFA (**10**)

Figure S41: ^1^H-NMR spectrum of **10** in DMSO-d6.

Figure S42: RP-HPLC chromatogram (220 nm) of **10**.

#### c(DLA^(6-Br)^Wp^(NMe)^K^(6-Br)^WRG) × 2 TFA (**11**)

Figure S43: ^1^H-NMR spectrum of **11** in DMSO-d6.

Figure S44: RP-HPLC chromatogram (220 nm) of **11**.

#### c(DLA^(6-Cl)^Wp^(NMe)^K^(6-Br)^WRG) × 2 TFA (**12**)

Figure S45: UV traces at 220nm and 280 nm of **12**.

#### c(DLA^(6-Cl)^Wp^(NMe)^K^(Az)^ARG) × 2 TFA (**13**)

Figure S46: 1H-NMR-spectrum of **13** in DMSO-d_6_

Figure S47: ^13^C-NMR-spectrum of **13** in DMSO-d_6_

#### c(DLA^(6-Br)^Wp^(NMe)^K^(Az)^ARG) × 2 TFA (**14**)

Figure S48: ^1^H-NMR-spectrum of **14** in DMSO-d_6_

Figure S49: ^13^C-NMR-spectrum of **14** in DMSO-d_6_

Figure S50: UV traces at 220nm and 280 nm **14**.

#### c(DLA^(6-(4-vinyl-benzoate)^Wp^(NMe)^KFRG) × 2 TFA (**15**)

Figure S51: UV traces at 220nm and 280 nm of **15**.

#### c(DLA^(6-(9-anthracenyl)^Wp^(NMe)^KFRG) × 2 TFA (**16**)

Figure S52: UV traces at 220nm and 280 nm of **16**.

#### c(DLA^(5-(1-pyrenyl)^Wp^(NMe)^KFRG) × 2 TFA (**17**)

Figure S53: ^1^H-NMR spectrum of **17** in DMSO-d6.

Figure S54: RP-HPLC chromatogram (220 nm) of **17**.

#### c(DLA^(5-(3-aminophenyl)^Wp^(NMe)^KFRG)× 3 TFA (**18**)

Figure S55: ^1^H-NMR spectrum of **18** in DMSO-d6.

Figure S56: RP-HPLC chromatogram (220 nm) of **18**.

#### c(DLA^(5-Cl)^Wp^(NMe)^K^(6-Br)^WRG) × 2 TFA (**19**)

Figure S57: ^1^H-NMR-spectrum of **19** in DMSO-d_6_

Figure S58: ^13^C-NMR-spectrum of **19** in DMSO-d_6_

Figure S59: UV traces at 220nm and 280 nm of **19**.

#### c(DLAFp^(NMe)^K^(6-(9-anthracenyl)^WRG) × 2 TFA (**20**)

Figure S60: UV traces at 220nm and 280 nm of **20**.

#### c(DLA^(6-Cl)^Wp^(NMe)^K^(6-(9-anthrancenyl)^WRG) × 2 TFA (**22**)

Figure S61: UV traces at 220nm and 280 nm of **22**.
